# Heterologous vaccination interventions to reduce pandemic morbidity and mortality: Modeling the US winter 2020 COVID-19 wave

**DOI:** 10.1073/pnas.2025448119

**Published:** 2022-01-11

**Authors:** Nathaniel Hupert, Daniela Marín-Hernández, Bo Gao, Ricardo Águas, Douglas F. Nixon

**Affiliations:** ^a^Department of Population Health Sciences, Weill Cornell Medicine, New York, NY 10065;; ^b^Division of General Internal Medicine, Department of Medicine, Weill Cornell Medicine, New York, NY 10065;; ^c^Cornell Institute for Disease and Disaster Preparedness, Cornell University, New York, NY 10065;; ^d^Division of Infectious Diseases, Department of Medicine, Weill Cornell Medicine, New York, NY 10065;; ^e^Centre for Tropical Medicine and Global Health, Nuffield Department of Medicine, University of Oxford, Oxford OX3 7BN, United Kingdom

**Keywords:** COVID-19, vaccination, trained immunity, infectious disease modeling, BCG

## Abstract

COVID-19 remains a stark health threat worldwide, in part because of minimal levels of targeted vaccination outside high-income countries and highly transmissible variants causing infection in vaccinated individuals. Decades of theoretical and experimental data suggest that nonspecific effects of non–COVID-19 vaccines may help bolster population immunological resilience to new pathogens. These routine vaccinations can stimulate heterologous cross-protective effects, which modulate nontargeted infections. For example, immunization with *Bacillus* Calmette–Guérin, inactivated influenza vaccine, oral polio vaccine, and other vaccines have been associated with some protection from SARS-CoV-2 infection and amelioration of COVID-19 disease. If heterologous vaccine interventions (HVIs) are to be seriously considered by policy makers as bridging or boosting interventions in pandemic settings to augment nonpharmaceutical interventions and specific vaccination efforts, evidence is needed to determine their optimal implementation. Using the COVID-19 International Modeling Consortium mathematical model, we show that logistically realistic HVIs with low (5 to 15%) effectiveness could have reduced COVID-19 cases, hospitalization, and mortality in the United States fall/winter 2020 wave. Similar to other mass drug administration campaigns (e.g., for malaria), HVI impact is highly dependent on both age targeting and intervention timing in relation to incidence, with maximal benefit accruing from implementation across the widest age cohort when the pandemic reproduction number is >1.0. Optimal HVI logistics therefore differ from optimal rollout parameters for specific COVID-19 immunizations. These results may be generalizable beyond COVID-19 and the US to indicate how even minimally effective heterologous immunization campaigns could reduce the burden of future viral pandemics.

## Significance

Control of the COVID-19 pandemic has been impeded by the slow global uptake of targeted vaccines, emergence of more transmissible variants, and resistance to continuation of nonpharmaceutical interventions. Commonly used vaccines can have nonspecific immune effects, and several have been shown to have beneficial heterologous effects against SARS-CoV-2 infection. However, there is no science-based guidance on effective implementation of such heterologous vaccine interventions (HVIs) to counter the current or future pandemics. We modeled the effect of different HVI strategies on the winter 2020 COVID-19 wave in the United States, finding that targeting both elderly and nonelderly populations and intervening during pandemic growth phases (i.e., effective reproduction number > 1) led to the greatest reduction in morbidity and mortality.

On March 16th, 2020, Imperial College London released a landmark report advocating the suppression of SARS-CoV-2 to avoid a pandemic catastrophe (1). Since then, the scientific community has been challenged to create a “bridge period” of reduced COVID-19 morbidity and mortality until safe and effective targeted vaccines are delivered globally (2). Guided by major modeling groups and international and national public health authorities, most countries quickly implemented variably stringent nonpharmaceutical interventions (NPIs) including physical distancing, self-isolation, home working, school closure, and “shielding” of vulnerable populations such as the elderly. Despite ameliorating COVID-19 incidence when applied, these “lockdowns” of regional and national economies also caused severe financial, social, and health repercussions globally (3). In the United States and other countries, resistance to and reversal of NPIs occurred in many jurisdictions, complicating pandemic control and contributing to persistently high COVID-19 incidence.

The rollout of specific COVID-19 vaccines in 2021 led to a temporary reduction of pandemic caseloads in countries with effective vaccine campaigns and ample stocks, but even this has not proven to be the sought-for panacea for epidemiological, logistical, and political reasons. The emergence of virus variants—now dominated by the Omicron and Delta strains—that are more transmissible and pathogenic have reversed many gains achieved to date and have raised questions about the durability of current vaccine efficacy (4). Although a handful of mainly high-income countries have instituted vigorous campaigns that have rapidly provided high coverage, less than 5% of the world’s low-income population has received at least one COVID-19–specific vaccination (5), and even in countries with ample vaccine supply, the global phenomenon of multifactorial vaccine hesitancy has led to uneven intranational uptake that has been exploited by the Delta variant. For these reasons, the public health armamentarium against COVID-19 has ample room for adjuncts to both NPIs and COVID-19–specific vaccines.

One as-yet unutilized intervention to potentially prevent SARS-CoV-2 infection and reduce COVID-19 disease is based upon heterologous or nonspecific effects (NSEs) induced by available non–SARS-CoV-2 vaccines (6). The heterologous effect of vaccination refers to the impact that vaccines can have on unrelated infections and diseases. These effects have been noted for almost a hundred years (7), and potential mechanisms include innate and adaptive immune responses. Trained immunity (8–14), increased cytokine production (15–17), viral interference (18), long-lasting type I interferons (19), the antiviral state (20), cross-reactivity (21, 22), and bystander activation (23) are some of the mechanisms proposed.

Some of the best-studied heterologous vaccine actions are from “off-target effects” from the *Bacillus* Calmette–Guérin (BCG) vaccine (12, 24–29). Epidemiological evidence including several randomized controlled trials (RCTs) have assessed the effect of BCG vaccination on reducing neonatal mortality. In Guinea-Bissau, two RCTs of BCG given to low weight neonates showed reduction in neonatal mortality after BCG, mainly because of fewer cases of neonatal sepsis, respiratory infection, and fever (30, 31). A meta-analysis of three RCTs of BCG-Denmark showed a reduction in mortality rate of 38% at 28 d of life; marked reductions in mortality were also seen within 3 d after vaccination and at 12 mo of age (32). Interestingly, a BCG vaccination prior to an influenza vaccination can boost influenza-specific immunity (33).

Because of the nonspecific benefits of BCG vaccination, a phase III trial called “ACTIVATE-2” assessed whether BCG could protect against COVID-19 in the elderly; prepublication findings suggest a 68% risk reduction for total COVID-19 clinical and microbiological diagnoses (34). A separate study showed that a history of BCG vaccination was associated with a decreased SARS-CoV-2 seroprevalence across a diverse cohort of healthcare workers, and reduced COVID-19 symptoms (35). The magnitude of protective effect against symptomatic disease was similar in both studies: a reported range of 10 to 30% reduction in all respiratory infections in the former and a 34.5% reduction in self-reported diagnosis of COVID-19 in the latter.

Other epidemiological studies have shown NSE benefits from oral polio vaccine (OPV), measles-containing vaccines (MCVs), and several other common immunizations. OPV has been associated with beneficial NSE (20, 36–38) and may become pronounced with subsequent doses (39–41). A systematic review of the associations of BCG, diptheria-tetanus-pertussis, and MCVs with childhood mortality showed that BCG and MCVs reduced overall mortality by more than would be expected through their effects on the diseases they target (42). As with BCG, an RCT of MCV showed a beneficial nonspecific effect on children’s survival (43).

Focusing on SARS-CoV-2 transmission, several studies have found that the administration of OPV, *Hemophilus* influenza type-B, measles mumps rubella (MMR), varicella, hepatitis A/B, pneumococcal conjugate, and inactivated influenza vaccines are associated with decreased SARS-CoV-2 infection rates (44–46). In addition, results from a study in a Dutch hospital showed a 37 to 49% lower risk of SARS-CoV-2 infection in healthcare workers who received the influenza vaccine in the previous flu season, and this finding was also corroborated by a preliminary in vitro study (9). Thus, there is some evidence to support an impact of routine vaccinations on SARS-CoV-2 infection rates, although these ecological studies are prone to bias, do not establish causality, and may be SARS-CoV-2 variant-specific.

Vaccine-mediated heterologous effects could also extend to reducing the severity of COVID-19 disease. There are epidemiological associations between those who have had a past vaccination with BCG, MMR, inactivated influenza vaccine, and recombinant adjuvanted zoster vaccine and reduced mortality and/or reduced COVID-19 severity (35, 45, 47–57), although these additional ecological studies are similarly susceptible to bias. A recent interim analysis of an ongoing clinical trial in Brazil supports this claim, showing that vaccination with MMR reduces the risk of COVID-19 symptoms and need for treatment (58). Given that the COVID-19 pandemic is still a global health emergency (especially in undervaccinated countries) and that the premise of HVI is soundly based in the immunological and epidemiological literature, there is ample reason to consider its potential role as part of a comprehensive package of pandemic control strategies.

The plethora of studies cited can help characterize the hypothetical efficacy of immune system boosting through HVIs to reduce COVID-19 morbidity and mortality. However, estimating the potential real-world effectiveness of such interventions requires their implementation in an environment that can quantify their potential population-level impact in the context of ongoing control measures on viral transmission, health care utilization, and health outcomes. This type of epidemiological projection can be achieved through the use of mathematical models of infectious disease (59–65).

We used the COVID-19 International Modeling (CoMo) Consortium Model (https://comomodel.net), an open-source, age-structured, country-specific, dynamic compartmental model of SARS-CoV-2 transmission and COVID-19 illness, treatment, and mortality, to illustrate how the logistics of implementing a heterologous vaccine intervention (HVI)—in particular, the timing of initiation of such a vaccination campaign in relation to trends in disease incidence and also the age-related population targeting of such a campaign—largely determine the magnitude of their impact. In particular, we instituted an explicitly defined HVI in one of three distinct time frames during the large fall/winter wave of SARS-CoV-2 in the United States (presurge, intrasurge, and postsurge) and across the same total number of individuals in one of three distinct age-targeted population groups (20+ y old, 40+ y old, and 65+ y old).

There are multiple potential applications of heterologous vaccination in this setting, e.g., as a pre–COVID-19 vaccination primer, as a simultaneously delivered or post–COVID-19 vaccination booster (i.e., replacing or delaying the use of a second COVID-specific vaccine dose), or as a solitary “bridging” intervention to reduce or delay COVID-19-related morbidity and mortality until a specific vaccine is available. Here, we explore the last use: that of a solo heterologous vaccination used as a temporizing “bridging” intervention that has only a low level of heterologous effectiveness at reducing viral transmission (here defined as reducing the likelihood of being infected by 5, 10, or 15%) and clinical severity (i.e., reducing the risk of death if infected, again by 5, 10, or 15%). Given the high levels of targeted vaccination now attained in many high-income countries, our results with respect to the prevaccinated US outbreak should be seen as general, model-informed operational guidance that could maximize the beneficial effect of efforts to use common vaccination programs to mitigate and temporize the impact of COVID-19, and possibly future viral pandemics, in the majority of countries worldwide that have not yet received sufficient quantities of COVID-19–specific vaccines to ensure population protection.

## Results

**Fig. 1.**
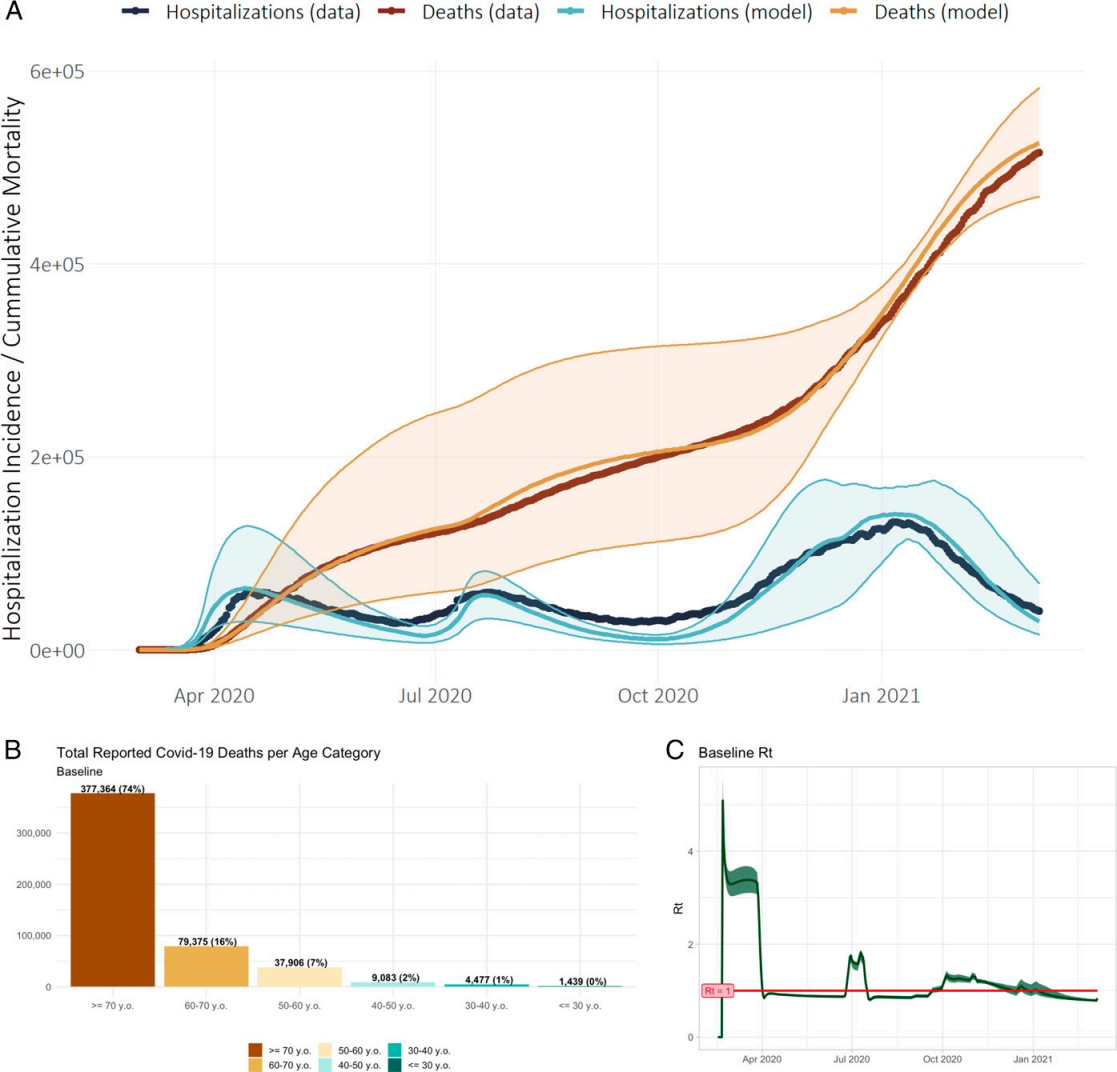
(*A–C*) (*A*) Comparison of CoMo Model output to reported COVID-19–related non-ICU hospitalizations and mortality in the United States of America, February 16th, 2020 through March 7th, 2021 (median prediction ± 95% CI with 0.03 SD Gaussian “noise” applied to 24 key model variables); (*B*) age-specific mortality and (*C*) effective reproduction number (Rt) for full simulation February 16th, 2020, through June 30th, 2021.

Through March 7th, 2021, there were 29,034,160 reported SARS-CoV-2 infections and 524,652 COVID-19 deaths in the US. From March 17th, 2020 (when reporting started), through March 7th, 2021, the US saw an average of 56,405 daily COVID-19 non-intensive care unit (ICU) hospitalizations. Fig. 1*A* shows the fit of the baseline CoMo Model USA simulation with this reported cumulative mortality and daily hospitalization, while Fig. 1 *B* and *C* show the age-stratified mortality and calculated reproductive number (Rt) through the full simulation from February 16th, 2020, to June 30th, 2021. Through March 7th, 2021, the baseline simulation predicts a mean of 28,366,733 reported cases [95% credible interval (CI) 15,137,456 to 45,664,234 across 100 simulations]. The model also projects 525,097 deaths attributed to COVID-19 (95% CI 469,852, 582,443) and a mean of 49,766 non-ICU hospitalizations (27,159, 79,476), including 17,594 (9,821, 27,879) ICU admissions with and without mechanical ventilation. Approximately three-fourths of all deaths occur in the 70+ age group, and 99% occur in individuals 40 y old and older. Rt, the effective reproduction number of the outbreak, rises above 1 from June 26th to July 17th and then again September 22nd through December 3rd, 2020. The baseline model outputs for cases, hospitalizations, and mortality are within 2.5, 1, and 12% of actual publicly available totals for the US COVID-19 outbreak through March 7th, 2021, respectively.*

**Fig. 2.**
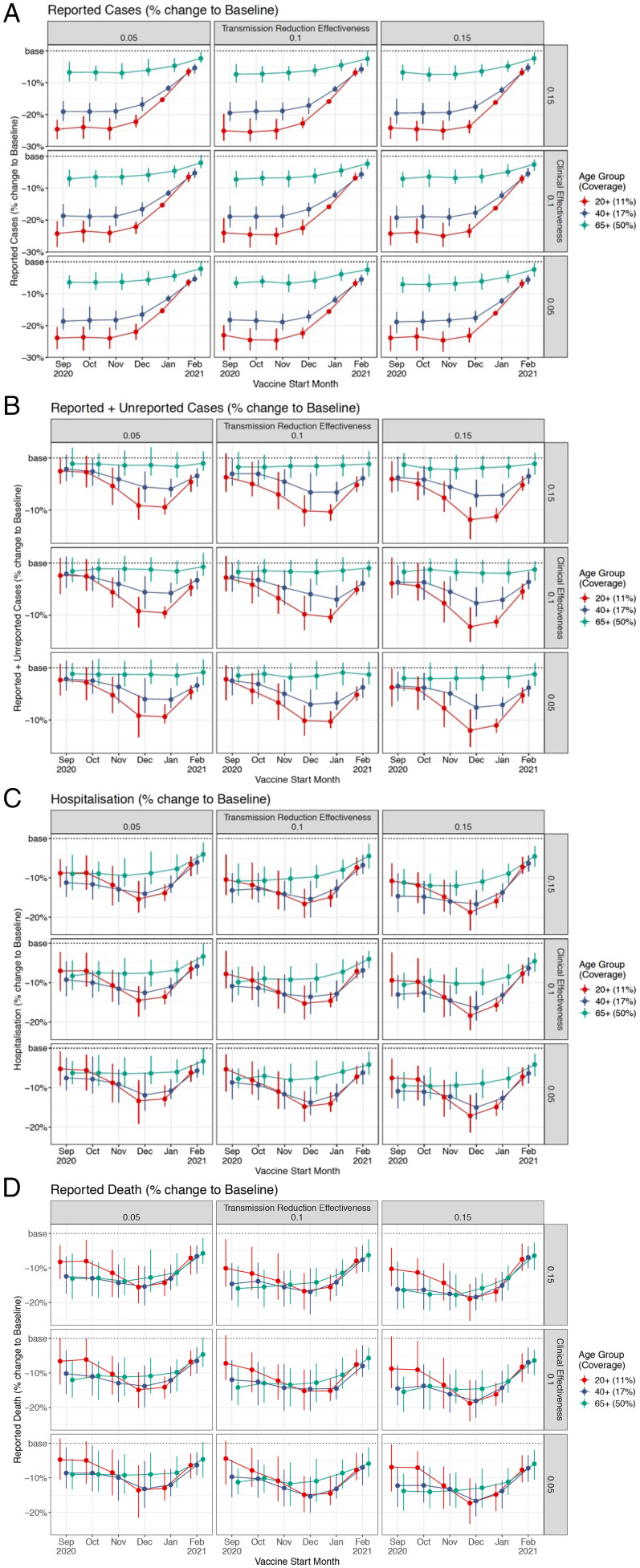
(*A–D*) Results of modeled HVI on reported cases (*A*), reported and unreported cases (*B*), hospitalization (*C*), and reported death (*D*) varying hypothetical effectiveness at reducing disease transmission (different columns) and clinical severity of disease (different rows), month of campaign initiation (horizontal axis), and population age threshold (11% of 20+ y olds in red, 17% of 40+ y olds in blue, and 50% of 65+ y olds in green).

Introduction of logistically realistic heterologous vaccination campaigns of different efficacy (reducing viral transmission and, independently, disease severity by 5, 10, or 15%) that target three different age-defined populations (fractions of the 20+, 40+, and 65+ age groups, with each cohort containing the same final number of vaccinated adults) at different timepoints in during the US Fall 2020 COVID-19 wave led to different simulated epidemiological and clinical outcomes (Fig. 2 *A–D*). Across the range of heterologous vaccine effectiveness, “early” interventions (September 1st and October 1st, 2020, prior to or during the onset of the Fall 2020 surge) lead to the largest decline in reported COVID-19 cases (that is, cases that are clinically symptomatic and therefore more likely to be detected; Fig. 2*A*; *Materials and Methods*). In contrast, later interventions lead to a larger reduction in both reported and unreported cases, with the greatest effect seen in interventions targeting the broadest targeted age group (20+ y old) starting in December and January (Fig. 2*B*). Similarly, COVID-19 hospitalization and mortality were most reduced with interventions that begin November 1st and December 1st, 2020, during which time the Rt rose to remain persistently greater than 1 (Fig. 1*C*), signaling an accelerating pandemic surge. To summarize, across the range of HVI efficacy (5 to 15%), applying the intervention to the broadest age-defined population (in this case, ∼27 million, or 11%, of the US population age 20 and older, compared to 17% of the US population age 40 and older or 50% of the US population age 65 and older, cohorts that also include ~27 million each) during the height of the pandemic surge (that is, with vaccination campaigns commencing December 1st) led to the greatest reductions in total cases, hospitalizations, and mortality compared to the baseline simulation (Table 1). This resulted in a model projection of 477,700 (95% CI ± 22,300) deaths for high- and 510,700 (95% CI ± 26,500) for low-efficacy heterologous vaccines versus a baseline of 591,800 through June 30th, 2021, without an HVI (meaning mortality reductions of 19 ± 4% and 16 ± 5%, respectively). For hospitalizations and reported cases, the corresponding percentage reductions are 24 ± 1% and 22 ± 1% for high-efficacy HVI and 19 ± 3% and 13 ± 3% for low-efficacy HVI, respectively.

**Table 1.** Mean reduction in reported cases, hospitalization, and reported deaths or mortality from equal-sized HVIs by targeted age group, assuming maximal 15% efficacy for both transmission reduction and clinical effectiveness

HVI initiation month	20+			40+			65+		
	Reported cases	Hospitalization	Mortality	Reported cases	Hospitalization	Mortality	Reported cases	Hospitalization	Mortality
September	24.1	7.9	7.3	18.9	10.9	12.7	6.8	9.1	13.3
October	24.3	9.0	8.4	18.9	11.3	12.4	6.8	9.0	13.1
November	**24.5**	12.1	11.8	18.8	12.8	14.1	6.7	9.1	13.2
December	22.6	**16.0**	**16.4**	17.0	14.2	15.7	6.0	8.7	12.5
January	15.7	14.4	14.9	11.9	12.2	13.5	4.5	7.4	10.7
February	6.7	7.0	7.4	5.4	6.3	7.0	2.3	4.0	5.7

Percent reduction versus baseline simulation through June 30th, 2021; maximal reduction in each category in bold.

**Fig. 3.**
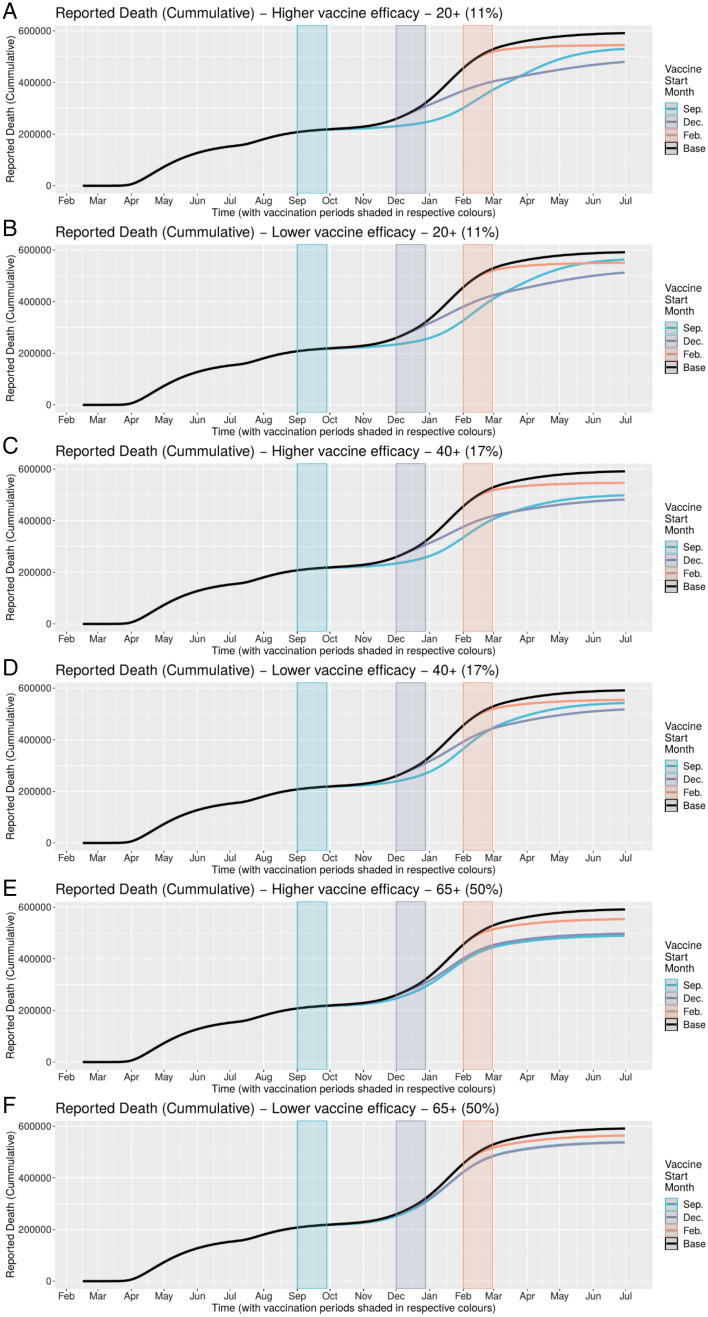
(*A–F*) Cumulative mortality under different HVI scenarios varying age targeting and timing of initiation. HVI initiation in December (when Rt > 1) yields greatest reduction in cumulative mortality across all combinations of HVI population age targeting and heterologous vaccine effectiveness.

**Fig. 4.**
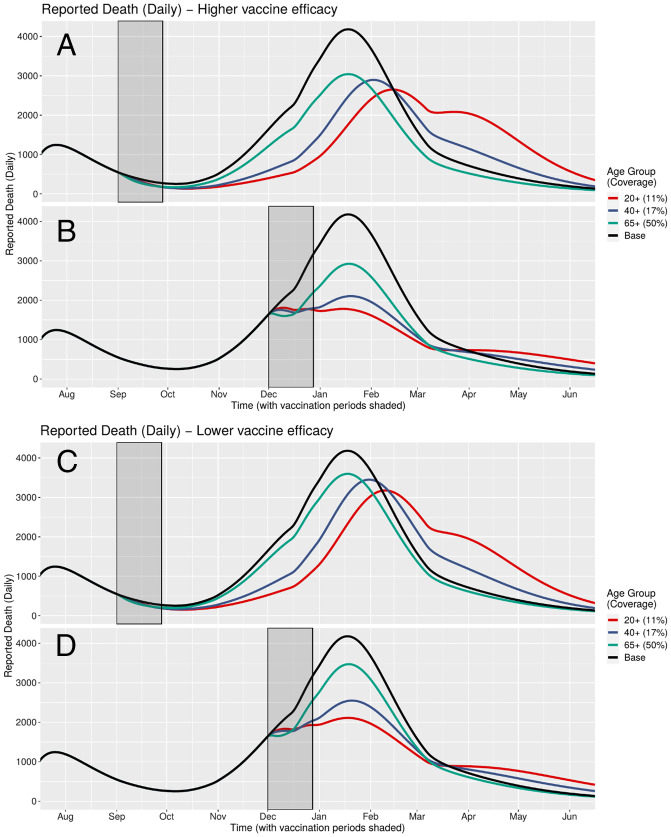
(*A–D*) Daily mortality under different HVI scenarios varying both age targeting and timing of initiation (but holding constant the total number of vaccinations across scenarios). HVI targeting the widest age range (11% of 20+ y olds) that is initiated during pandemic surge (Rt > 1) yields the greatest reduction in peak mortality at both high and low heterologous vaccine effectiveness.

Cumulative (Fig. 3 *A–F*) and daily (Fig. 4 *A–D*) mortality graphs illustrate how the magnitude of HVI’s mortality-reduction effects vary with the timing of its implementation in relation to the pre-, intra-, and postsurge periods, corresponding to Rt < 1, Rt > 1, and Rt < 1. As best shown in the daily figures, initiation of HVI in the presurge period delays the rise in reported deaths in inverse relation to the targeted age threshold (with younger targeting leading to greater delay but with comparatively similar peak mortality). In contrast, initiation of HVI during the onset of the surge appears to arrest the momentum of the rise in mortality more abruptly, again in inverse proportion to the targeted age threshold, with a greater differentiation of peak mortality than in the timing of that peak. These effects are qualitatively similar for high and low HVI effectiveness scenarios.

**Fig. 5.**
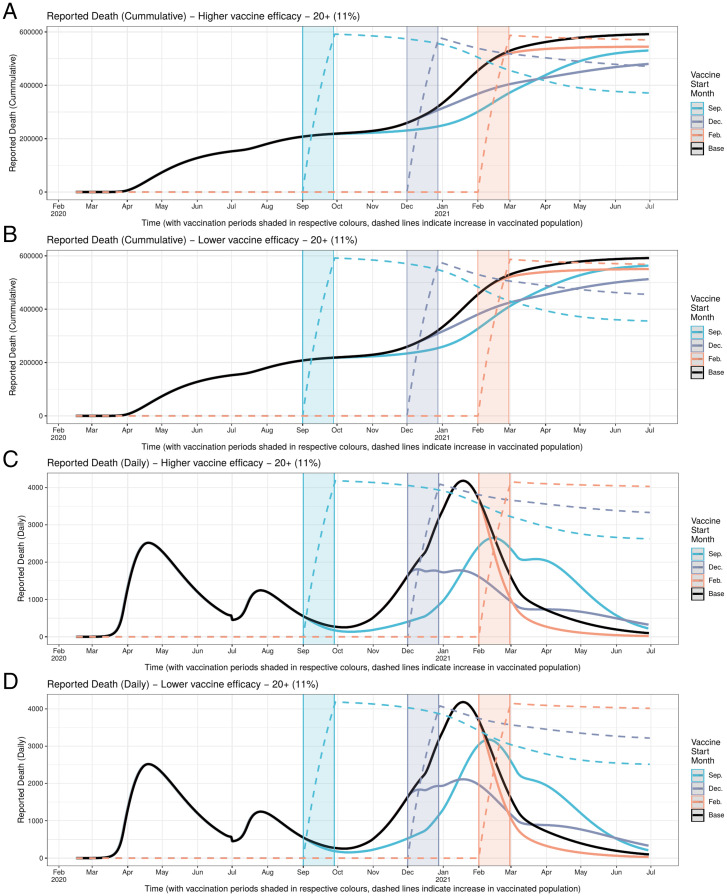
(*A–D*) Dynamic interaction of heterologous vaccination coverage of 11% of USA population 20 y old and older, showing attenuation of heterologously vaccinated population pool and both cumulative and daily projected reported deaths under three different HVI campaign start times (September, December, and February).

These effects are further illustrated in Fig. 5 *A–D*, which detail the rise (over 4 wk) and subsequent attenuation of heterologously vaccinated individuals for the 20+ age-group simulations with both high and low HVI efficacy. The decline in number of these susceptible and partially protected individuals (dashed lines) is due to modeled COVID-19 infection and is rapid enough that there are fewer remaining uninfected September vaccinees when that epidemic curve peaks (roughly in early February) than there are remaining uninfected December vaccinees when that curve peaks (in mid-December). This helps to explain why the December HVI start leads to superior outcomes versus the September start, since there are relatively fewer susceptible individuals available with the former intervention when the force of infection is highest.

**Fig. 6.**
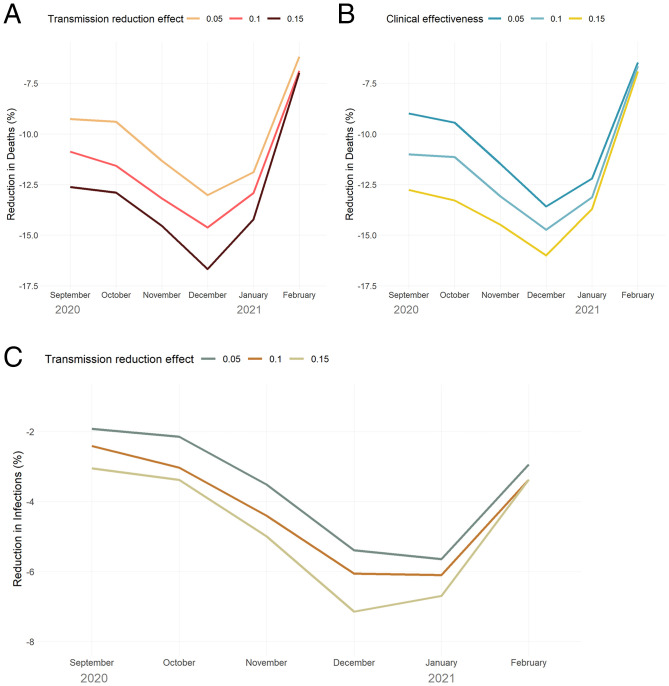
(*A–C*) Reduction in mortality (*A*, *B*) and infections (*C*) at different levels of HVI efficacy, varying the timing of initiation of HVI campaign. *A* and *B* illustrate the differential impact of efficacy in reducing viral transmission versus efficacy in reducing clinical severity of disease (i.e., preventing hospitalization and risk of death); *C* illustrates how even a constant (15%) heterologous efficacy in transmission reduction leads to widely varying levels of infection reduction based on HVI timing.

Fig. 6 *A–C* summarizes the mortality and disease transmission impact of various levels and types of HVI efficacy (i.e., transmission reduction versus clinical effectiveness) for the 20+ age cohort experiments. Three findings emerge: first, that there is a nonlinear relationship between hypothetical HVI efficacy and modeled effectiveness that is highly dependent on timing of the intervention; second, that at higher efficacy levels, transmission reduction is more effective than clinical benefit (i.e., reducing disease severity) at reducing deaths; and third, that the mortality benefit conferred by transmission reduction is roughly double its effect in reducing the number of infections. Further details of these temporal effects are discussed in *SI Appendix, Experimental Parameters and Baseline Results*; full model outputs for the baseline and December 1st, 2020, HVI campaign targeting 20+ y olds is available at https://github.com/ocelhay/como.

## Discussion

Using the US experience of COVID-19 through Spring 2021 as a modeling “test bed,” we show that the addition of even minimally efficacious HVIs to ongoing NPIs may reduce pandemic caseloads, hospitalization, and mortality. However, our simulations reveal that the effectiveness of such heterologous interventions depends heavily on several key operational parameters, including the timing of HVI implementation in relation to pandemic disease activity and also the age targeting of the HVI campaigns. Contrary to guidance for the phased rollout of COVID-19–specific vaccines (e.g., from the US Advisory Committee on Immunization Practices [ACIP]: https://www.cdc.gov/vaccines/hcp/acip-recs/vacc-specific/covid-19.html), we find that targeting HVI campaigns to both elderly and nonelderly populations (i.e., those 20 y and older) and initiating those campaigns during pandemic growth phases (i.e., when Rt > 1) leads to the greatest reduction in COVID-19 morbidity and mortality.

Our results show notable improvement in important clinical outcomes generated by even low-effectiveness HVI interventions (i.e., those with clinical protection and transmission reduction of 5%) undertaken during the upswing of this surge. Furthermore, even low-effectiveness HVIs undertaken prior to such surges (whose timing may be highly unpredictable) may delay peak hospitalizations and mortality sufficiently to give time for specific vaccination campaigns to gain traction and prevent these delayed cases from presenting. In the specific US COVID-19 scenario modeled here, such interventions delay peak mortality from early January (at which point <10 million people had received at least one dose of specific COVID-19 vaccines) to mid-February (by which time >40 million had received at least one dose), creating a “bridging intervention” that potentially could have preserved hospital capacity, saved lives, and also prevented nonhospitalized cases that risk evolution into “Long COVID,” with its attendant health risks.

Our model realistically differentiates the likelihood of case finding on the basis of symptomaticity. Since younger individuals are less likely to be symptomatic when infected with SARS-CoV-2, changes in their infection status can be obscured with looking solely at reported cases. The greater decline in combined reported and unreported cases (Fig. 2*B*) compared to reported cases alone (Fig. 2*A*) when instituting HVI with broad age targeting is largely due to this lower rate of symptomaticity among younger infected individuals. This reduction in unreported cases among the nonelderly may be one critical factor in reducing spread to older individuals and therefore reduction in overall, population-wide mortality with this approach.

Our finding of improved overall outcomes when targeting vaccination to include these younger populations, even for a disease whose prime morbidity and mortality falls on older populations, has been found in modeling-based studies of both COVID-19 and other viral illnesses such as influenza (66, 67). Additionally, network-based modeling (which is methodologically distinct from the compartmental modeling framework used here) has found that targeting more “linked” individuals, rather than simply older people, optimally reduces COVID-19 morbidity and mortality for older and younger individuals alike (67). Interaction between simulated individuals in the CoMo Model is governed by the contact matrices generated by Prem et al. (68), who found that for high-income countries such as the USA, 20 to 45 y olds have the greatest number of off-axis (i.e., not with similar-aged) contacts. Interrupting disease transmission across these interactions with HVIs that reduce viral transmission, which may reduce infections in more clinically vulnerable older individuals, may help explain why the degree of mortality reduction exceeds the relative reduction in cases in our experiments with the broadest age targeting. As of this writing, non–high-income countries worldwide are grappling with the consequences of low rates of targeted vaccination for COVID-19. Since these countries tend to have relatively greater off-axis contacts among younger age groups, it is likely that these results apply outside of the US setting.

Model-based epidemiological studies such as ours are subject to many caveats, not least that models that are fitted to past data may not give accurate projections of future trends. To avoid predictive errors of this sort, we deliberately chose to perform retrospective “what if”-type simulations of different HVIs in a well-fitted modeling environment that could accommodate a realistic suite of NPIs in combination with experimental interventions and could recreate in silico the course of the US pandemic experience through the start of widespread specific COVID-19 vaccination. This is typically called “scenario” or “intervention modeling” as opposed to forecasting. Although this framework is amenable to modeling the use of heterologous vaccination as an adjunct or extender of such specific vaccination campaigns (e.g., replacing a second dose of a two-shot regimen with a heterologous booster), there is little evidence available to parameterize such an effect. Even with the HVI modeled here, the choice of the 5 to 15% range of clinical protection and transmission reduction is admittedly based on limited and largely indirect evidence (69). For this reason, we conservatively chose to model a maximal effect size that is half of that reported in the current literature (35, 70). We assume that heterologous effects extend through and do not attenuate during the experimental time frame of interest, which ranges from 4 to 10 mo. If these effects do wane, our findings would overstate the effectiveness of HVIs.

A concluding thought is that HVIs may be useful for "flattening the curve" of the current pandemic in at least two ways: by delaying and reducing peak COVID-19 transmission in the majority of low- and middle-income countries that, because of lack of vaccine availability or other factors have yet to fully mount COVID-19–specific vaccine campaigns; and by lowering infection rates and consequent hospitalizations and deaths in both minimally and highly vaccinated nations during surges of vaccine-escaping SARS-CoV-2 variants such as Omicron (for which, paradoxically, "wild type"-directed COVID-19–specific vaccines may have HVI-like effects similar to the ones explored here). Since they would be limited to the use of already fully licensed vaccines, HVIs should not carry increased risks for targeted populations beyond those of standard vaccination campaigns, and therefore may help circumvent delays and outright resistance related to concerns about the safety of novel pandemic-targeting vaccines. Because typically they would involve the use of widely used vaccines, to which a large portion of the population has been antigenically sensitized, HVIs may provide rapid immune boosting for immediate viral threats (i.e., more rapid than the 3+ wk required for novel targeted vaccines to generate robust immunological responses). For these reasons, we believe that HVIs should take their place alongside NPIs in the “early pandemic intervention toolkit” of public health emergency response planners, forming part of the public health armamentarium against both inevitable future pandemics and breakthrough infection risk from more transmissible or immune-escaping variants of current pathogens. In addition to their solely health-related impact, these interventions could also help to mitigate the severe social and economic effects of lockdown policies through earlier pandemic control. If increased uptake of nonspecific vaccination, alone or in combination with other routinely recommended vaccine interventions, could partially attenuate the COVID-19 pandemic, it may provide public health and political leaders with sufficient gains around which to leverage increased social and economic activity to reverse some of the devastating toll this infection has taken on daily life worldwide.

## Materials and Methods

We used the publicly available COVID-19 CoMo Consortium Model (version 18.1, https://comomodel.net) to carry out the simulations reported here. The CoMo Model, developed to support policy-related analyses of potential COVID-19 management strategies by Consortium members (https://como.bmj.com), is an open-source, spreadsheet template–based, age-structured, compartmental susceptible-exposed-infectious-recovered-susceptible model to estimate the trajectory of COVID-19 based on different scenarios and assess the potential impact of various nonpharmaceutical/behavioral change strategies as well as treatment and vaccines. Details of the modeling framework, including all NPI settings and other parameter values used in the current simulations, are available in the *SI Appendix*; an overview of the model structure, including all mathematical equations governing progression through clinical and health care utilization compartments, has been published elsewhere (see SI Appendix of Águas et al.; ref. 71).

The CoMo Model is distinguished from standard age-structured compartmental models by several attributes: It is country specific (in terms of both population structure and age-based mixing); it incorporates detailed disease severity–specific treatment compartments as well as explicit health care facility utilization based on the Cornell COVID Caseload Calculator (https://phs.weill.cornell.edu/research-collaboration/divisions-institutes/cornell-institute-disease-disaster-preparedness/covid); it can be parameterized to cases, mortality, hospitalizations, or any combination of these three for a defined region; and it can implement one or more of nine NPIs (handwashing, mask wearing, full or partial school closure, self-isolation, social distancing, shielding of the elderly, mass testing, and working at home), one medical intervention (dexamethasone), and logistically realistic vaccination campaigns (with specified start dates and ramp-up periods, as well as adjustable age targeting). Because the model is freely available on the Web and is template based, anyone can both recreate and extend the experiments reported here using the information supplied in the *SI Appendix*.

Model calibration was achieved by adjusting parameters relating to viral activity (e.g., start of modeling window and probability of transmission given encounter), hospitalization and mortality (i.e., age-specific risk of hospitalization and hospital mortality rate based on Salje; ref. 72), temporally adjusted NPIs [including handwashing, mask wearing, social distancing, self-isolation, working at home, shielding of the elderly, and international travel bans, all based in part on Google Mobility reports (https://www.google.com/covid19/mobility/)], and treatment (e.g., institution of dexamethasone treatment for patients requiring supplemental oxygen in late summer 2020). Data on total US COVID-19 cases and mortality were obtained from the New York Times GitHub site (https://raw.githubusercontent.com/nytimes/covid-19-data/master/us.csv), and hospitalization data were obtained from The COVID Tracking Project (https://covidtracking.com/data/national/hospitalization/) through March 7th, 2021.

All simulations were run with identical slates of NPIs instituted in the USA, including increased handwashing, variable mask wearing, social distancing, self-isolation of the ill, international travel bans, school closure, and shielding of the elderly; in addition, we factored in the introduction of dexamethasone therapy starting in the second half of summer 2020. Experiments were run using three vaccination campaign age thresholds (20+, 40+ or 65+ y old), six start dates (vaccination campaigns starting the first day of each month from September 2020 through February 2021), and three levels of heterologous vaccine efficacy both for reducing SARS-CoV-2 transmission and for subsequent risk of COVID-19 hospitalization and mortality if infected (set at 5, 10, or 15% for each parameter). Due to the brief (∼1 y) time frame of interest, there was no temporal waning of heterologous effects.

Based on reported rates of seasonal influenza vaccination coverage among the general population across US states ranging from 37.8 to 63.4% (US Centers for Disease Control and Prevention, 2017 data, https://www.cdc.gov/flu/fluvaxview/nursinghome/report1718/reporti/index.html), we anchored HVI coverage in the 65+ age cohort at 50%, representing ∼27.4 million people. To ensure that the number of individuals vaccinated in each of the three targeted populations was identical (i.e., ∼27 million), we adjusted the vaccination campaigns to target 11% of those 20 older, 17% of those 40 and older, and 50% of those 65 and older in the US population. This eliminated the possibility of bias resulting from relatively more or fewer resultant heterologous vaccinees in the three age-targeted population cohorts. The timing of interventions coincided with typical annual influenza vaccination (i.e., September through February), which in 2020 also coincided with the presurge, intrasurge, and postsurge period of the nationwide fall/winter US COVID-19 wave.

The baseline model was calibrated to US COVID-19 case, mortality, and hospitalization data through March 7th, 2021, with inflation of predicted cases to match hospitalization and death rates prior to July 2020 when the US testing first reached sufficient coverage to achieve a national positivity rate <5% (ref. 73; see *SI Appendix* for further details on modeling framework and parameters.) The mean incubation period was 3.5 d, and the mean duration of the infectious period was 4.5 d, each with a range of 1 to 7 d). Mean hospital lengths of stay were modeled as 6 d for regular medical ward, 10 d for ICU stay without mechanical ventilation, and 12 d for duration of mechanical ventilation, consistent with both published literature and the US-based Epic Health Research Network (https://epicresearch.org/articles/inpatient-lengths-of-stay-and-number-of-icu-days-among-covid-19-patients-differ-from-common-model-assumptions/). Total modeled US hospital, ICU, and mechanical ventilatory capacity (800,685 and 60,327 beds and 60,000 ventilators, respectively) was never exceeded during any simulation run. We assumed that 75% of all symptomatic and 2% of all asymptomatic SARS-CoV-2 infections would be reported, 15% of all infected individuals would develop severe enough symptoms to warrant hospitalization, 25% of hospitalized patients required ICU admission, 60% of those admitted to the ICU required mechanical ventilation, and 50% of all hospitalized patients require supplemental oxygen (see *SI Appendix* for details). Targeted COVID-19 vaccination commenced in the US on December 14th, 2020, and increased from that time onwards to the end of the modeled period (June 30th, 2021). To account for the effect of COVID-19 vaccination, we relaxed modeled NPI controls after this point to appropriately fit mortality and hospitalizations. In this way, although COVID-19 vaccination was not explicitly modeled, its effect on population outcomes is incorporated into all results.

The baseline model was calibrated to the data by ensuring the data falls within the 95% model credible intervals generated from 100 model iterations applying 0.03 SD Gaussian “noise” to the 24 most sensitive model parameters (see *SI Appendix* for complete parameterization information). The model was run from February 16th, 2020, through June 30th, 2021. Each of the 162 experimental model outputs were generated from 50 iterations for each distinct parameter and intervention set subjected to 0.01 SD “noise” to generate 95% credible intervals. Statistical analysis was performed with the R statistical package (version 3.6.3) and Microsoft Excel 2016 (Redmond, WA).

## Supplementary Material

Supplementary File

## Data Availability

Complete code for the CoMo Model and outputs of the baseline and December HVI initiation experiments are freely available at the COVID-19 International Modeling (CoMo) Consortium GitHub site: https://github.com/ocelhay/como and https://github.com/ocelhay/como/blob/master/misc/pnas/PNAS_MS_2020-25448_Supplemental_Data.csv.

## Footnotes

*Given the likelihood that reported COVID-19–related mortality rates have undercounted its true impact in most jurisdictions, the modeled fit to reported deaths was given lower priority than fit to hospitalizations and cases.
