# Supplementary material for: Heterologous vaccination interventions to reduce pandemic morbidity and mortality: Modeling the US winter 2020 COVID-19 wave
Source: Proc Natl Acad Sci U S A. 2022 Jan 11;119(3):e2025448119. doi: 10.1073/pnas.2025448119 (PMC8784160; doi:10.1073/pnas.2025448119)
Supplement: Supplementary File [file pnas.2025448119.sapp.pdf]

Modeling heterologous vaccination interventions (HVI) to reduce pandemic hospitalization and mortality: a study of USA's winter 2020 COVID-19 wave

Supplementary Materials:

Modeling Framework and Mathematical Model…………………………………….2

Experimental Parameters and Baseline Results…………………………………….18

Disclaimer for CoMo Consortium Model……………………………………………….36

1. Modeling Framework and Mathematical Model

The CoMo Consortium SARS-CoV-2 model is an age-structured SEIR model with infected compartments stratified by symptoms, severity and treatment seeking and access. The progression of individuals through the infection life cycle is represented by the diagram below.

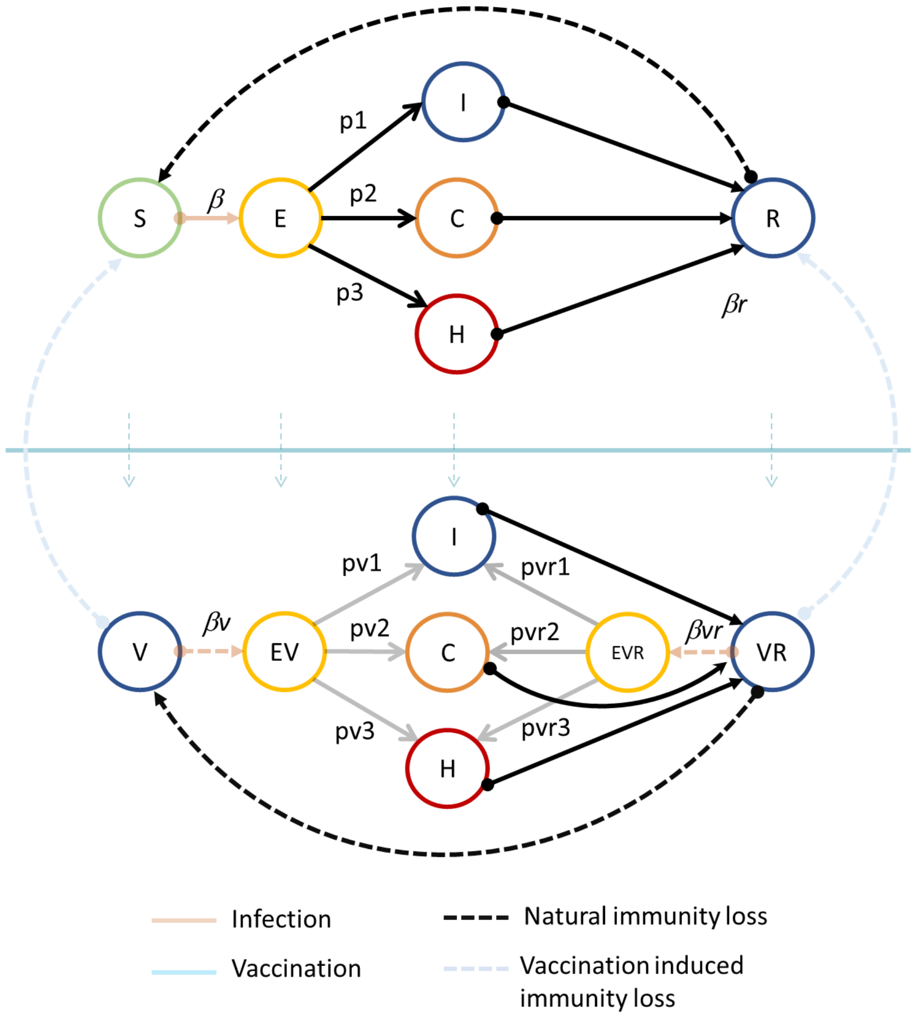

$$\{\beta, p1, p2, p3\} \neq \{\beta v, pv1, pv2, pv3\}$$

$$\{\beta, p1, p2, p3\} \neq \{\beta r, \beta vr, pvr1, pvr2, pvr3\}$$

Figure S1: A diagram of the baseline model structure including vaccination and a collapsed hospital sub model.

As susceptible individuals (*S*) are infected, they transit through an incubation phase, *E*, at the end of which they are fully infectious. At the end of this incubation period, individuals can display very different symptomatology, with some never having any symptoms (*A*), others having minor symptoms (*C*), and fewer requiring hospitalization (*H*). If a person is hospitalized, the model tracks their health care requirements and treatment cascades until they are release from hospital or die. Disease-induced mortality rates are heavily dependent on how severe the infection outcome is and whether individuals seek to/get appropriate treatment. The cascade of treatment seeking, care requirements and hospital stress factors resulting in what treatment the patient ends up getting relative to their needs is summarized in Figure S2.

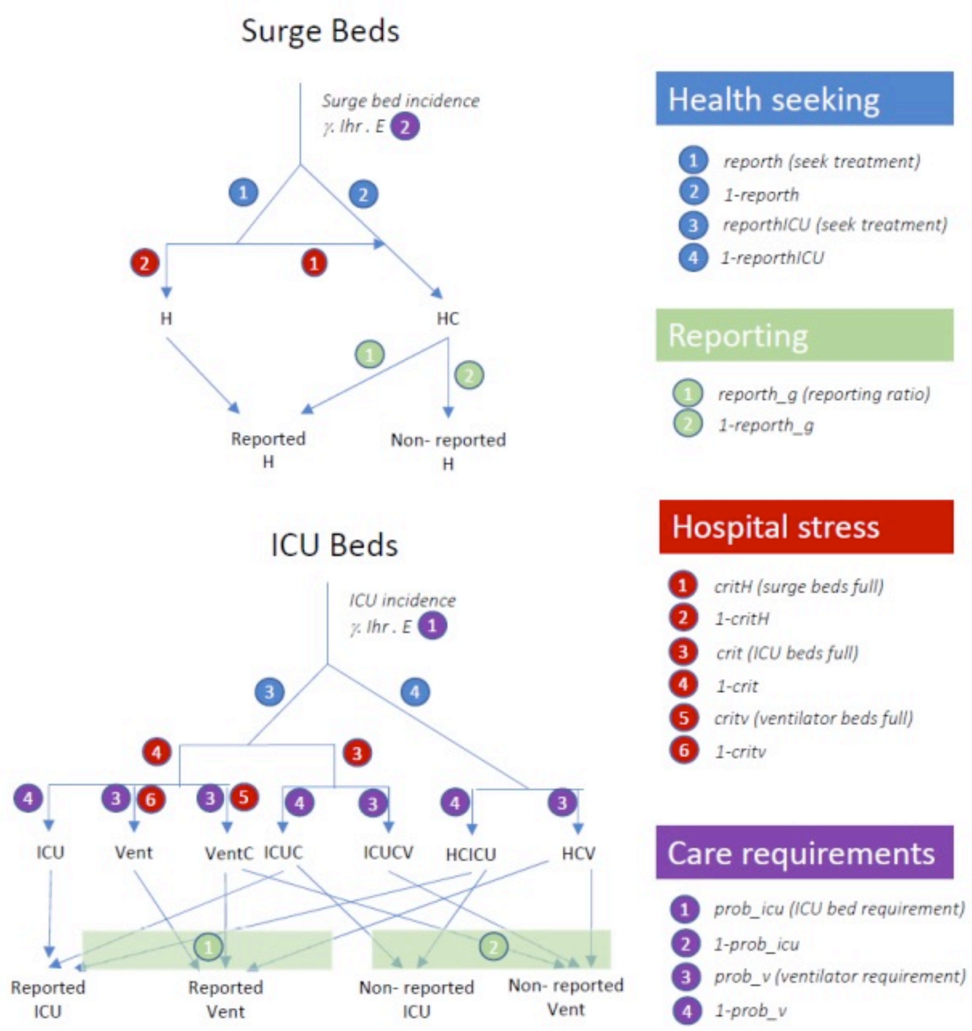

Figure S2. Schematic representation of the hospital sub model.

Importantly, several reporting rates can be defined to account for health surveillance systems' inefficiencies in capturing the correct epidemiological metrics. There are a total of 14 reporting rates that could inform the local idiosyncrasies and describe the appropriate context for each model use case. The model assumes that those who recover from infection will become immune, although this protection state can be transient as we allow for loss of immunity following both natural infection and vaccination (dashed lines in Figure S1).

The equations for the basic model structure without interventions follow:

$$\frac{dS}{dt} = -S \circ \Lambda + \omega R + A \cdot S - \mu \cdot S + bP$$

$$\frac{dE}{dt} = S \circ \Lambda - \gamma E + A \cdot E - \mu \cdot E$$

$$\frac{dI}{dt} = \gamma\,(1 - p_{clin})(1 - p_{ihr}) \cdot E - \nu_I I + A \cdot I - \mu \cdot I$$

$$\frac{dC}{dt} = \gamma\, p_{clin}(1 - p_{ihr}) \cdot E - \nu_I C + A \cdot C - \mu \cdot C$$

$$\frac{dR}{dt}$$

$$= \nu_I(I + C) + A \cdot R - \omega R - \mu \cdot R + \left(1 - \delta_H\, p_{hfr}\right)\nu_H \cdot H + \left(1 - \delta_{H_c}\, p_{hfr}\right)\nu_H \cdot H_c + \left(1 - \delta_U\, p_{hfr}\right)\nu_U \cdot U + \left(1 - \delta_{U_c}\, p_{hfr}\right)\nu_U \cdot U_c + \left(1 - \delta_{V_c}\, p_{hfr}\right)\nu_v \cdot U_{cv} + \left(1 - \delta_V\, p_{hfr}\right)\nu_V \cdot Vent + \left(1 - \delta_{V_c}\, p_{hfr}\right)\nu_V \cdot Vent_c$$

$$\frac{dH}{dt} = p_{ihr}(1 - p_U)(1 - p_{K_H})\gamma E - \nu_H H + A \cdot H - \mu \cdot H$$

$$\frac{dH_c}{dt} = p_{ihr}(1 - p_U)p_{K_H}\gamma E - \nu_H H_c + A \cdot H_c - \mu \cdot H_c$$

$$\frac{dU}{dt} = p_{ihr}p_U\left(1 - p_{K_U}\right)(1 - p_V)\gamma E - \nu_U U + A \cdot U - \mu \cdot U$$

$$\frac{dU_c}{dt} = p_{ihr}p_U p_{K_U}(1 - p_V)\gamma E - \nu_U U_c + A \cdot U_c - \mu \cdot U_c$$

$$\frac{dU_{cv}}{dt} = p_{ihr}p_U p_{K_U}p_V\gamma E - \nu_V U_{cv} + A \cdot U_{cv} - \mu \cdot U_{cv}$$

$$\frac{dVent}{dt} = p_{ihr}p_U\left(1 - p_{K_U}\right)\left(1 - p_{K_V}\right)p_V\gamma E - \nu_V Vent + A \cdot Vent - \mu \cdot Vent$$

$$\frac{dVent_c}{dt} = p_{ihr}p_U\left(1 - p_{K_U}\right)p_{K_V}p_V\gamma E - \nu_V Vent_c + A \cdot Vent_c - \mu \cdot Vent_c$$

$$P = (S + E + I + C + R + H + H_c + U + U_C + U_{Cv} + Vent + Vent_c)$$

$$s = 1 + a\cos\left(2\pi\frac{\left(t - \left(\frac{365.25\phi}{12}\right) + t_{in}\right)}{365.25}\right)$$

$$W = W_{home} + W_{work} + W_{school} + W_{other}$$

$$\Lambda = p\ s\ W \cdot \left(\frac{\rho E + I + C + \rho_s * (H + H_c + U + U_c + U_{cv} + Vent + Vent_c)}{P}\right)$$

$$A = \begin{pmatrix} \begin{pmatrix} -\alpha & 0 \\ \alpha & -\alpha \end{pmatrix} & \cdots & 0 \\ \vdots & \ddots \begin{pmatrix} -\alpha & 0 \\ \alpha & -\alpha \end{pmatrix} \ddots & \vdots \\ 0 & \cdots & \begin{pmatrix} -\alpha & 0 \\ \alpha & 0 \end{pmatrix} \end{pmatrix}$$

$$p_{K_H} = \left.\begin{matrix} 0\ for\ H < K_H \\ 1\ for\ H \geq K_H \end{matrix}\right\}$$

$$p_{K_U} = \left.\begin{matrix} 0\ for\ U < K_U \\ 1\ for\ U \geq K_U \end{matrix}\right\}$$

$$p_{K_V} = \left.\begin{matrix} 0\ for\ V < K_V \\ 1\ for\ V \geq K_V \end{matrix}\right\}$$

The model uses publicly available country-specific data to define the population structure,[1] as a model input. A description of the model variables can be found in Table S1 and a list with all parameters included in the full model (including interventions) is given in Table S2.

Interventions

Several non-pharmaceutical interventions can be included in the model alongside vaccination; any of these interventions can be switched on or off for specific periods of time, with some able to target specific age groups such as school closures and vaccination. Notably, intervention coverage can be defined for each of the intervention periods as illustrated in Figure S3.

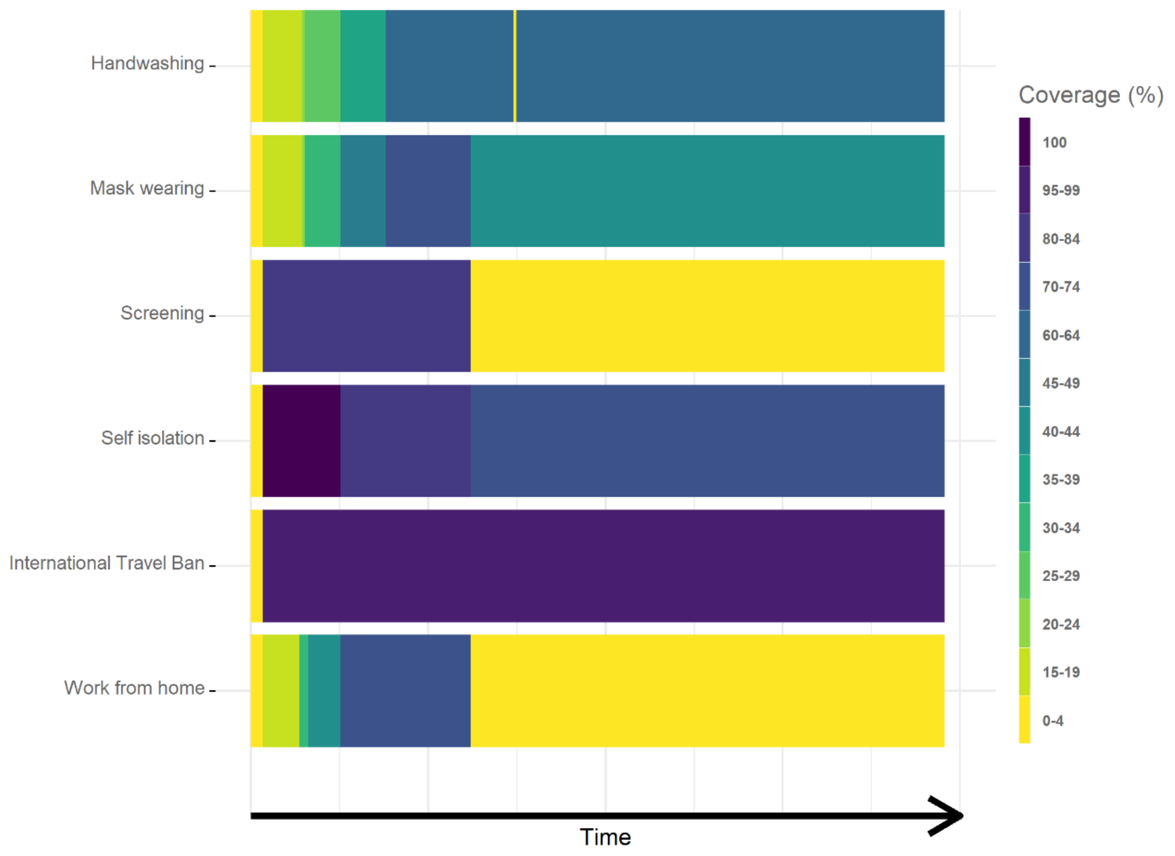

Figure S3. Illustration of a combination of modelled interventions, each with different coverage levels over time.

Self-Isolation if Symptomatic

This is the practice of individuals with either a confirmed case of Covid-19 or with Covid-19 symptoms isolating themselves at home for a period of 7 days. The parameters governing this intervention are:

- Start Date: the start date of the protocol
- Duration: the duration of the protocol
- Coverage: the percentage of the population who will be able to self-isolate if they have symptoms or are a confirmed case
- Adherence: the percentage of the designated isolation period that self-isolated individuals adhere to the intervention

Screening

This is a form of contact-tracing. Given enough testing capacity, it reflects how suspected contacts of confirmed cases are tested using a SARS-CoV-2 virological test. All individuals who test positive are then requested to self-isolate

- Start Date: the start date of additional screening
- Test Sensitivity: Probability that an infected person will test positive when screened
- Suspected Contacts: number of people screened per reported case
- Overdispersion: informs the probability of finding an infected person that is a known contact of a reported case, relative to random sampling (overdispersion = 1)
- Duration: duration of this additional protocol

Social Distancing

Also known as physical distancing, this refers to the measures taken to prevent the spread of a contagious disease by maintaining a specific physical distance between individuals and reducing the number of times individuals come into close contact with each other. The parameters governing this intervention are:

- Start Date: the start date of the protocol
- Duration: the duration of the protocol
- Coverage: the percentage of the population who reduce their societal contacts (excluding those at home, work and school)
- Adherence: the percentage of the time that those practicing physical distancing adhere to physical distancing measures

Handwashing

This indicates improvements in personal hygiene and reduction in risk behaviours (touching the face, nose or mouth), including the adoption of personal protective equipment such as masks. The parameters governing this intervention are:

- Start Date: the start date of the protocol
- Duration: the duration of the protocol
- Efficacy: the effectiveness of personal hygiene measures in reducing the risk of infection per contact

Working from Home

This indicates the effect of having workers working from home. The parameters governing this intervention are:

- Start Date: the start date of the protocol
- Duration: the duration of the protocol
- Efficacy: the percent reduction in work related contacts
- Home contacts inflation: the percent increase in the numbers of home contacts due to increased number of hours spent at home

School Closures

This indicates school closures and assumes that all schools in a country close at the same time. The parameters governing this intervention are:

- Start Date: the start date of the protocol
- Duration: the duration of the protocol
- Efficacy: defined as the percent reduction in contacts between school children when schools are closed
- Home contacts inflation: the percent increase in the numbers of home contacts due to increased numbers of hours spent at home
- Age groups affected by the school closure: list of age groups in five-year bins that stay at home. This can be re-defined for any time.

Shielding the Elderly

This intervention is designed to isolate a proportion of the elderly population and reduce their overall contacts. The parameters governing this intervention are:

- Start Date: the start date of the protocol
- Duration: the duration of the protocol
- Coverage: the percentage of the elderly population who are shielded
- Efficacy: defined as the percent reduction in overall contacts of the shielded elderly population
- Minimum age for elderly cocoon: the minimum age cut-off defining which people should protect themselves

International Travel Ban

This refers to a ban on international travel, by sea, land, or air. The parameters governing this intervention are:

- Start Date: the start date of the protocol
- Duration: the duration of the protocol
- Efficacy: the percent reduction in imported cases per day

Voluntary home quarantine

This indicates how many people will voluntarily quarantine themselves at home for a specified number of days if a person they live with tests positive for Covid-19. The parameters governing this intervention are:

- Start Date: the start date of the protocol
- Duration: the duration of the protocol
- Days in quarantine for an average person
- Coverage: The percentage of people voluntarily quarantining themselves given they live with a known infectious case
- Rate: Speed at which people decide to quarantine themselves if they live with a known infectious case
- Percent decrease in the number of other contacts when voluntarily quarantining: refers to decreased mean numbers of contacts outside of the home while quarantining
- Percent increase in the number of contacts at home when voluntarily quarantining: refers to increased numbers of home contacts due to increased time spent at home while quarantining

Mass Testing

We modelled mass testing campaigns by transforming a user defined input for the number of tests performed in the community per day (these are done in randomly selected people, which is the main difference relative to the screening option) into rates of isolation in governmental facilities. That is to say that given the number of tests performed per day, those testing positive will be taken to facilities where they will observe a quarantine period. The rate at which infected individuals are put in quarantine thus depends on the number of tests done per day and the likelihood that each test is done in an infected person. The parameters governing this intervention are:

- **Sensitivity: probability of an infected person testing positive if tested.**
- Isolation days: number of days people who test positive will remain in isolation.

Vaccination

Vaccination was designed to mimic vaccination campaigns in which different age groups are prioritized at different times. For each user defined vaccination period, a target coverage and the list of age groups (5 year age bins) of people to be vaccinated needs to be defined. Vaccination is implemented as a rate such that the target vaccine coverage in each vaccination campaign is reached at the end date of that period, as it would in reality. The parameters governing this intervention are:

- Vaccine coverage: target vaccine coverage per vaccination period.
- Target age groups. Can be any combination of the 21 age modelled age groups (in 5-year bins).
- Length of vaccination campaign: time to reach target vaccination coverage.
- Vaccine efficacy. This can be discriminated for each combination of infection outcome and prior exposure.
- Duration of efficacious period. This can be discriminated for different infection histories.

Table S1. Description of the model variables.

Symbol	Definition
S	Susceptible
E	Infected and incubating
I	Infectious and asymptomatic following incubation
C	Infectious and mildly symptomatic following incubation
R	Recovered and immune
H	Severe infection: hospitalised
H_c	Severe infection: not hospitalised due to lack of capacity
U	Severe infection: hospitalised in ICU
U_c	Severe infection: hospitalised and requiring ICU but placed in surge ward
U_{cv}	Severe infection: hospitalised and requiring ventilator but placed in surge ward
Vent	Severe infection: hospitalised in ICU and on a ventilator
$Vent_c$	Severe infection: hospitalised in ICU requiring a ventilator but not on one
V	Vaccinated
VR	Vaccinated with natural immunity
EV	Exposed after being vaccinated
EVR	Exposed with both vaccine and natural immunity

Table S2. A list of the default parameter values used. These are subject to change when the model is applied to a new setting and/or with new incoming information. We have provided references to demonstrate that the default values lie within plausible ranges. † Country-specific value; § Vaccine specific; ‡Assumed value (no reference found).

Symbol	Definition	Value	Unit	Source
**Demographics**				
$\boldsymbol{W_{home}}$	Country-specific age-dependent contact matrix describing the number of potentially infectious contacts at home per person per day	†	day^{-1}	[2]
$\boldsymbol{W_{work}}$	Country-specific age-dependent contact matrix describing the number of potentially infectious contacts at work per person per day	†	day^{-1}	[2]
$\boldsymbol{W_{school}}$	Country-specific age-dependent contact matrix describing the number of potentially infectious contacts at school per person per day	†	day^{-1}	[2]
$\boldsymbol{W_{other}}$	Country-specific age-dependent contact matrix describing the number of potentially infectious societal contacts per person per day	†	day^{-1}	[2]
$\boldsymbol{\mu}$	1/Age-dependent non-Covid-19-related death rate	†	days	[3]
$\boldsymbol{b}$	1/ Age-dependent fertility rate	†	days	[3]
$\boldsymbol{\alpha}$	Ageing rate between age categories	0.2	$year^{-1}$	
**Natural history of infection**				
$\boldsymbol{p}$	Probability of infection given a single contact	†	NA	[4]
$\boldsymbol{\gamma}$	1/duration of incubation period	3.5	days	[5-7]
$\boldsymbol{\rho}$	Relative infectiousness of incubating phase	0.1	NA	‡
$\boldsymbol{p_{clin}}$	Proportion of all infections that ever develop symptoms	0.55	NA	[8-10]
$\boldsymbol{\nu_I}$	1/duration of infectious phase post incubation	4.5	days	[5]

$\boldsymbol{\rho_s}$	Relative proportion of contacts for hospitalised patients	0.15	NA	‡
$\boldsymbol{\omega}$	1/duration of immunity	150	years	‡
**Seasonality**				
$\boldsymbol{a}$	Relative variation in viral transmissibility throughout the year (+- a proportion)	†	NA	-
$\boldsymbol{\phi}$	Month of peak in transmissibility	†	NA	-
**Patient outcomes**				
$\boldsymbol{p_{ihr}}$	Probability of an infection being severe (requiring hospitalisation) by age	†	NA	[3 11-14]
$\boldsymbol{p_{hfr}}$	Probability of a severe/hospitalised infection being fatal by age	†	NA	[3 11 12 14 15]
$\boldsymbol{\nu_H}$	1/Duration of hospitalised infection	†	days	[16]
$\boldsymbol{\nu_U}$	1/Duration of ICU infection	†	days	[17 18]
$\boldsymbol{\nu_V}$	1/Duration of ventilated infection	†	days	[5 6 15]
$\boldsymbol{\delta_H}$	Maximum probability of death for a hospitalised infection	0.35		[3 16]
$\boldsymbol{\delta_{H_c}}$	Maximum probability of death for an infection requiring hospitalisation that did not receive appropriate treatment	0.45	NA	[19]
$\boldsymbol{\delta_U}$	Maximum probability of death for a hospitalised infection requiring ICU admission	0.55	NA	[19 20]
$\boldsymbol{\delta_{U_c}}$	Maximum probability of death for a hospitalised infection that would require ICU admission but was not admitted to the ICU	0.8	NA	[19]
$\boldsymbol{\delta_V}$	Maximum probability of death for a hospitalised infection requiring a ventilator	0.8	NA	[19]

$\boldsymbol{\delta_{V_c}}$	Maximum probability of death for a hospitalised infection that would require a ventilator but did not get one	0.95	NA	[21]
$\boldsymbol{p_U}$	Probability of an infected patient needing ICU	0.5	NA	[13 21]
$\boldsymbol{p_V}$	Probability of an infected patient needing ICU and a ventilator	0.75	NA	[21]
$\boldsymbol{K_H}$	Standard hospital bed capacity	†	NA	-
$\boldsymbol{K_U}$	ICU bed capacity	†	NA	-
$\boldsymbol{K_V}$	Ventilator capacity	†	NA	-
**Reporting**				
**report_g**	Percentage of denied hospitalisations that are reported	†	NA	-
**report**	Percentage of all asymptomatic infections that are reported	†	NA	-
**reportc**	Percentage of all symptomatic infections that are reported	†	NA	-
**reporth**	Percentage of non-severe hospitalisations that are appropriately treated and reported	†	NA	-
**reporth_ICU**	Percentage of severe hospitalisations that are appropriately treated and reported	†	NA	-
**report_natdeathI**	Percentage of all people dying outside the hospital with asymptomatic infections reported as covid-deaths	†	NA	-
**report_natdeath CL**	Percentage of all people dying outside the hospital with symptomatic infections reported as covid-deaths	†	NA	-
**report_death_HC**	Percentage of all people dying outside the hospital with severe infections reported as covid-deaths	†	NA	-
**Vaccination**				

**pclin_v**	Probability upon infection of developing clinical symptoms if previously vaccinated	§	NA	-
**pclin_vr**	Probability upon infection of developing clinical symptoms if previously vaccinated and exposed	§	NA	-
**prob_icu_v**	Probability upon hospitalisation of requiring ICU admission if previously vaccinated	§	NA	-
**prob_icu_vr**	Probability upon hospitalisation of requiring ICU admission if previously vaccinated and exposed	§	NA	-
**prob_v_v**	Probability upon admission to the ICU of requiring a ventilator if previously vaccinated	§	NA	-
**prob_v_vr**	Probability upon admission to the ICU of requiring a ventilator if previously vaccinated and exposed	§	NA	-
**sigmaEV**	Probability of requiring hospitalization if previously vaccinated	§	NA	-
**sigmaEVR**	Probability of requiring hospitalization if previously infected and vaccinated	§	NA	-
**Vac_campaign**	Time to reach target coverage (in weeks)	§	NA	-
**Vac_dur**	Duration of efficacious period	§	NA	-
**Vac_dur_r**	Duration of efficacious period if previously infected	§	NA	-
**Vaccine_eff**	Efficacy against infection	§	NA	-

References

1. World Population Prospects 2019: United Nations; 2020 [Available from: https://population.un.org/wpp/Download/Standard/Population/.
2. Prem K, Cook AR, Jit M. Projecting social contact matrices in 152 countries using contact surveys and demographic data. *PLoS Comput Biol* 2017;13(9):e1005697. doi: 10.1371/journal.pcbi.1005697
3. COVID-19: Data 2020 [Available from: https://www1.nyc.gov/site/doh/covid/covid-19-data.page.
4. Otto MA. COVID-19 update: Transmission 5% or less among close contacts 2020 [Available from: https://www.the-hospitalist.org/hospitalist/article/218769/coronavirus-updates/covid-19-update-transmission-5-or-less-among-close.
5. Linton NM, Kobayashi T, Yang Y, et al. Incubation Period and Other Epidemiological Characteristics of 2019 Novel Coronavirus Infections with Right Truncation: A Statistical Analysis of Publicly Available Case Data. *J Clin Med* 2020;9(2) doi: 10.3390/jcm9020538 [published Online First: 2020/02/17]
6. Khalili, M., Karamouzian, M., Nasiri, N., Javadi, S., Mirzazadeh, A., & Sharifi, H. Epidemiological characteristics of COVID-19: a systematic review and meta-analysis. *Epidemiology and Infection* 2020, 148, e130. https://doi.org/10.1017/S0950268820001430
7. Bi Q, Wu Y, Mei S, et al. Epidemiology and transmission of COVID-19 in 391 cases and 1286 of their close contacts in Shenzhen, China: a retrospective cohort study. *Lancet Infect Dis.* 2020 Aug;20(8):911-919. doi: 10.1016/S1473-3099(20)30287-5. Epub 2020 Apr 27. Erratum in: Lancet Infect Dis. 2020 Jul;20(7):e148. PMID: 32353347; PMCID: PMC7185944.
8. Mizumoto K, Kagaya K, Zarebski A, et al. Estimating the asymptomatic proportion of coronavirus disease 2019 (COVID-19) cases on board the Diamond Princess cruise ship, Yokohama, Japan, 2020. *Euro Surveill.* 2020 Mar;25(10):2000180. doi: 10.2807/1560-7917.ES.2020.25.10.2000180. Erratum in: Euro Surveill. 2020 Jun;25(22): PMID: 32183930; PMCID: PMC7078829.
9. Day M. Covid-19: four fifths of cases are asymptomatic, China figures indicate. *BMJ* 2020;369:m1375. doi: 10.1136/bmj.m1375 [published Online First: 2020/04/02]
10. Coronavirus disease 2019 (COVID-19) pandemic: increased transmission in the EU/EEA and the UK – seventh update [Available from: https://www.ecdc.europa.eu/sites/default/files/documents/RRA-seventh-update-Outbreak-of-coronavirus-disease-COVID-19.pdf.
11. Vital Surveillances: The Epidemiological Characteristics of an Outbreak of 2019 Novel Coronavirus Diseases (COVID-19) — China, 2020 [Available from: http://weekly.chinacdc.cn/en/article/id/e53946e2-c6c4-41e9-9a9b-fea8db1a8f51.
12. Hauser A, Counotte MJ, Margossian CC, et al. Estimation of SARS-CoV-2 mortality during the early stages of an epidemic: A modeling study in Hubei, China, and six regions in Europe. *PLoS Med*. 2020 Jul 28;17(7):e1003189. doi: 10.1371/journal.pmed.1003189. PMID: 32722715; PMCID: PMC7386608.

13. Verity R, Okell LC, Dorigatti I, et al. Estimates of the severity of coronavirus disease 2019: a model-based analysis. *Lancet Infect Dis* 2020 doi: 10.1016/s1473-3099(20)30243-7 [published Online First: 2020/04/03]
14. Coronavirus: summary of persons being monitored, persons under investigation, and cases [Available from: https://floridadisaster.org/globalassets/covid19/dailies/covid-19-data---daily-report-2020-04-25-0956.pdf.
15. ICNARC report on COVID-19 in critical care [Available from: https://www.icnarc.org/DataServices/Attachments/Download/913f4820-704c-ec11-9139-00505601089b].
16. Zhou F, Yu T, Du R, et al. Clinical course and risk factors for mortality of adult inpatients with COVID-19 in Wuhan, China: a retrospective cohort study. *Lancet* 2020;395:1054-62. doi: 10.1016/S0140-6736(20)30566-3 [published Online First: 2020/03/11]
17. Wang Z, Ji JS, Liu Y, et al. Survival analysis of hospital length of stay of novel coronavirus (COVID-19) pneumonia patients in Sichuan, China. *medRxiv* 2020:2020.04.07.20057299. doi: 10.1101/2020.04.07.20057299
18. Huang C, Wang Y, Li X, et al. Clinical features of patients infected with 2019 novel coronavirus in Wuhan, China. *Lancet* 2020;395(10223):497-506. doi: 10.1016/s0140-6736(20)30183-5 [published Online First: 2020/01/28]
19. Petrilli CM, Jones SA, Yang J, et al. Factors associated with hospital admission and critical illness among 5279 people with coronavirus disease 2019 in New York City: prospective cohort study. *BMJ* 2020; 369 :m1966 doi:10.1136/bmj.m1966
20. Lewnard JA, Liu VX, Jackson ML, et al. Incidence, clinical outcomes, and transmission dynamics of severe coronavirus disease 2019 in California and Washington: prospective cohort study. BMJ. 2020 May 22;369:m1923. doi: 10.1136/bmj.m1923. Erratum in: BMJ. 2020 Jun 4;369:m2205. PMID: 32444358; PMCID: PMC7243800.
21. Richardson S, Hirsch JS, Narasimhan M, et al. Presenting Characteristics, Comorbidities, and Outcomes Among 5700 Patients Hospitalized With COVID-19 in the New York City Area. *JAMA* 2020 doi: 10.1001/jama.2020.6775 [published Online First: 2020/04/22]

2. Experimental Parameters: Summary of Model Simulations

The following is a summary of the output from the CoMo Consortium model version v18.1.0 which can be accessed through an online interface at https://comomodel.net/.

Model Calibration

Inputs

The CoMo model uses demographic data for Country from UN 2019 Revision of World Population Prospects (`https://population.un.org/wpp/Download/Standard/Population/`). The model is age structured and the contacts between age classes are assumed to follow those predicted in [1] for United States of America. Cases and mortality data are downloaded from the `European Centre for Disease Prevention and Control website`. Severity and risk of death are assumed to be age dependent and default values follow estimates from France [2].

The simulation was started on 2020-02-16 with the following interventions assumed to be applied for the duration of the observed data: *Handwashing, Mask Wearing, Social Distancing, Self-isolation if Symptomatic, Working at Home, International Travel Ban, School Closures, Shielding the Elderly, Dexamethasone.*

The following chart illustrates the timelines of these interventions:

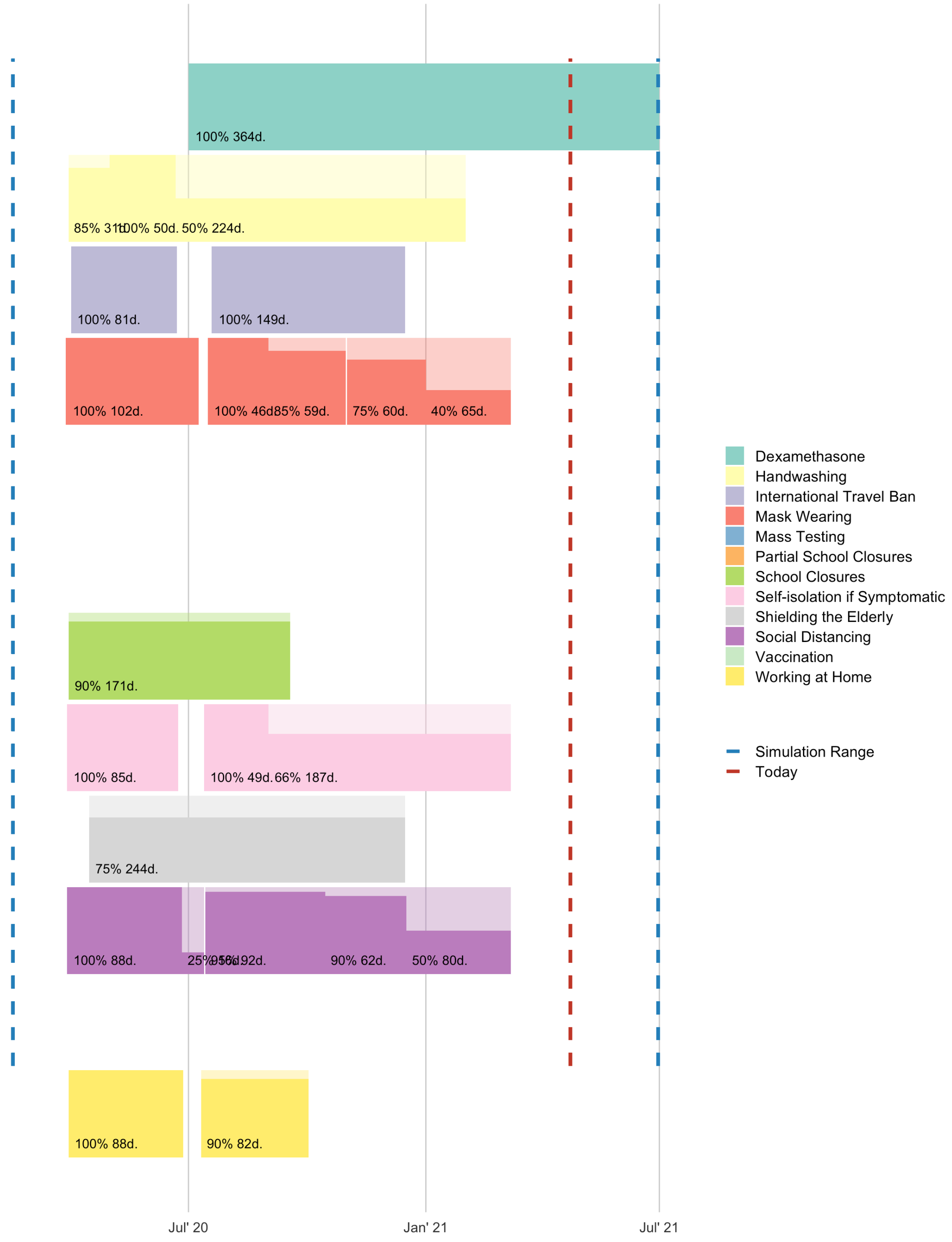

There follows a list of the key input values (for a full list, please see the Appendix):

- Probability of infection given a contact is 0.031
- Percentage of all asymptomatic infections reported is 2%
- Percentage of all symptomatic infections reported is 75%

- Percentage of all hospital surge bed occupancy (in the app: of all hospitalisations) reported is 90%

Model output compared with data

The following graph shows the reported cases data compared with the model prediction:

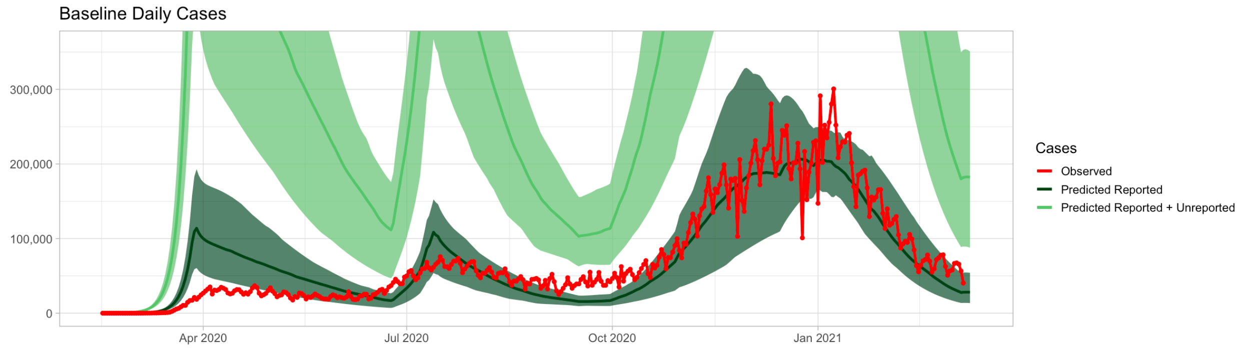

The following graph shows the cumulative mortality data compared with the model prediction:

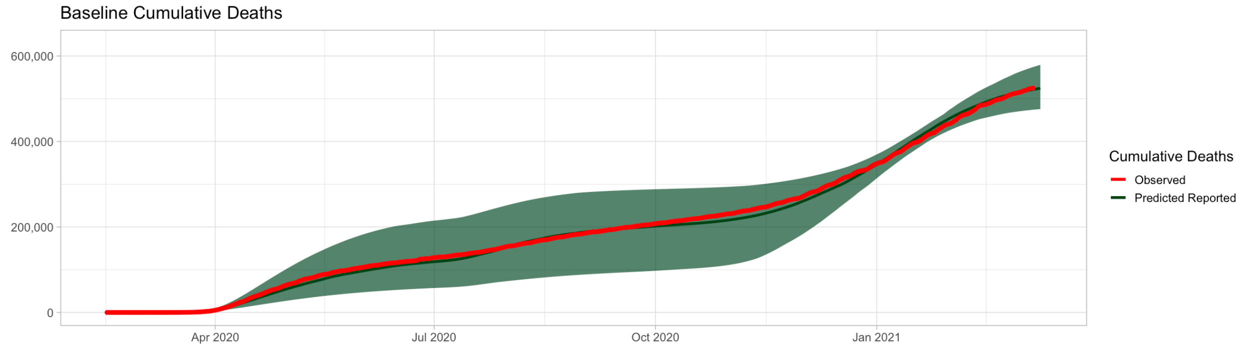

The following graph shows the modeled daily hospital census data:

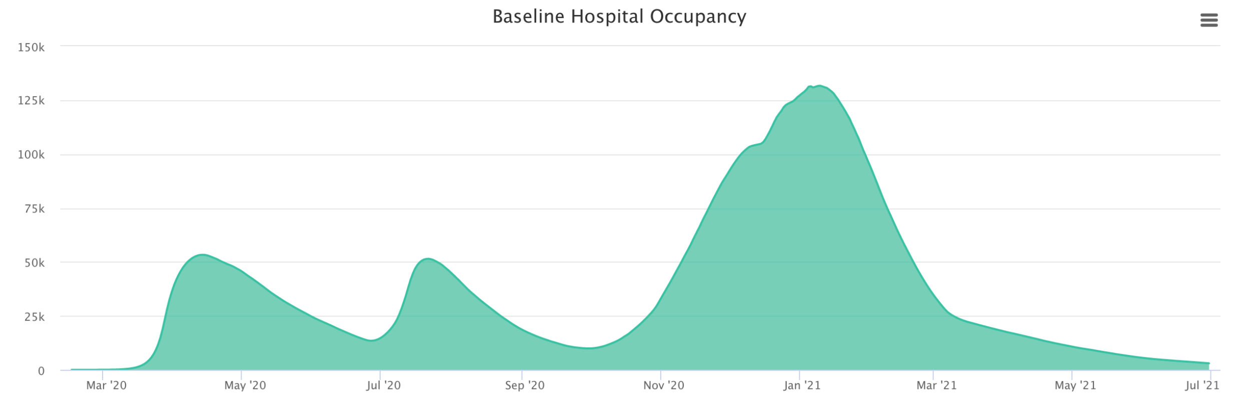

For this "baseline scenario" between 2020-02-16 and 2021-06-30:

- 62% (95% credible interval: 58.9, 65.9) of the population is predicted to have experienced the infection (including unreported, mildly symptomatic, and/or asymptomatic infections)
- 627,906 (95% credible interval: 355,137, 984,761) Covid-19 attributable deaths are expected during the range of simulation

Baseline compared with hypothetical scenario

The following series of charts represents a series of model projections for various values over time for both baseline and hypothetical scenarios. In reviewing this output, note that COVID-19 is a novel disease. Knowledge on transmission dynamics is still being discovered. Models are therefore based on assumptions and unknown information about the disease. Model inputs and outputs will change as we learn more about the disease and the impact of interventions on the disease. Uncertainty is even more pronounced in a country or population where the epidemic is still at early stage. Options and outputs will change once better serology, treatments, vaccines become widely available.

A "hypothetical scenario" was simulated with the same inputs values except for the interventions which were set up as follows: *Handwashing, Mask Wearing, Social Distancing, Self-isolation if Symptomatic, Working at Home, International Travel Ban, School Closures, Shielding the Elderly, Dexamethasone, Vaccination.*

The following chart illustrates the timelines of these interventions:

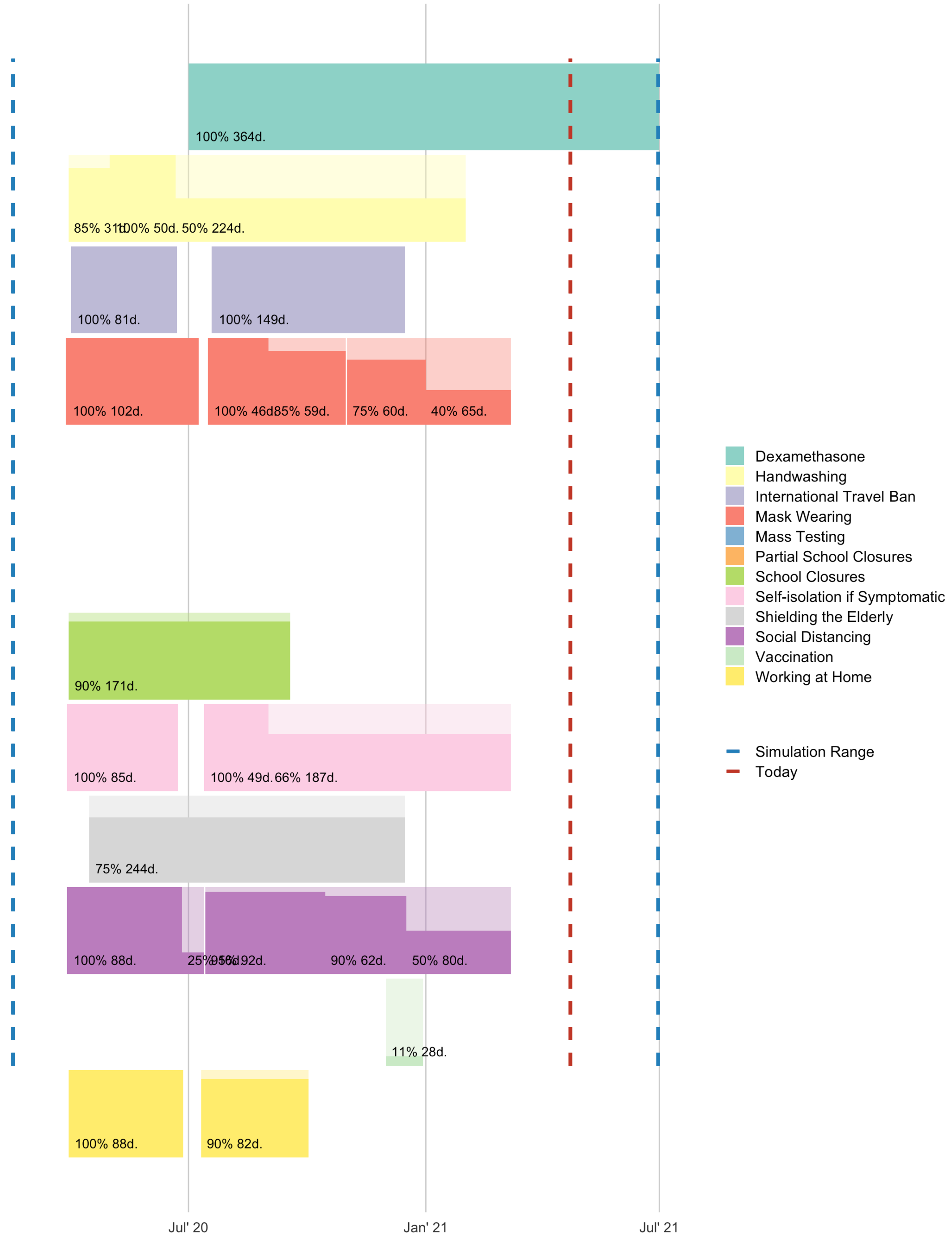

For this "hypothetical scenario" between 2020-02-16 and 2021-06-30:

- 56.75% (95% credible interval: 51.7, 62.2) of the population is predicted to have experienced the infection (including unreported, mildly symptomatic, and/or asymptomatic infections)
- 556,237 (95% CI: 269,675, 914,071) Covid-19 attributable deaths are expected during the range of simulation

Predicted Reported + Unreported cases

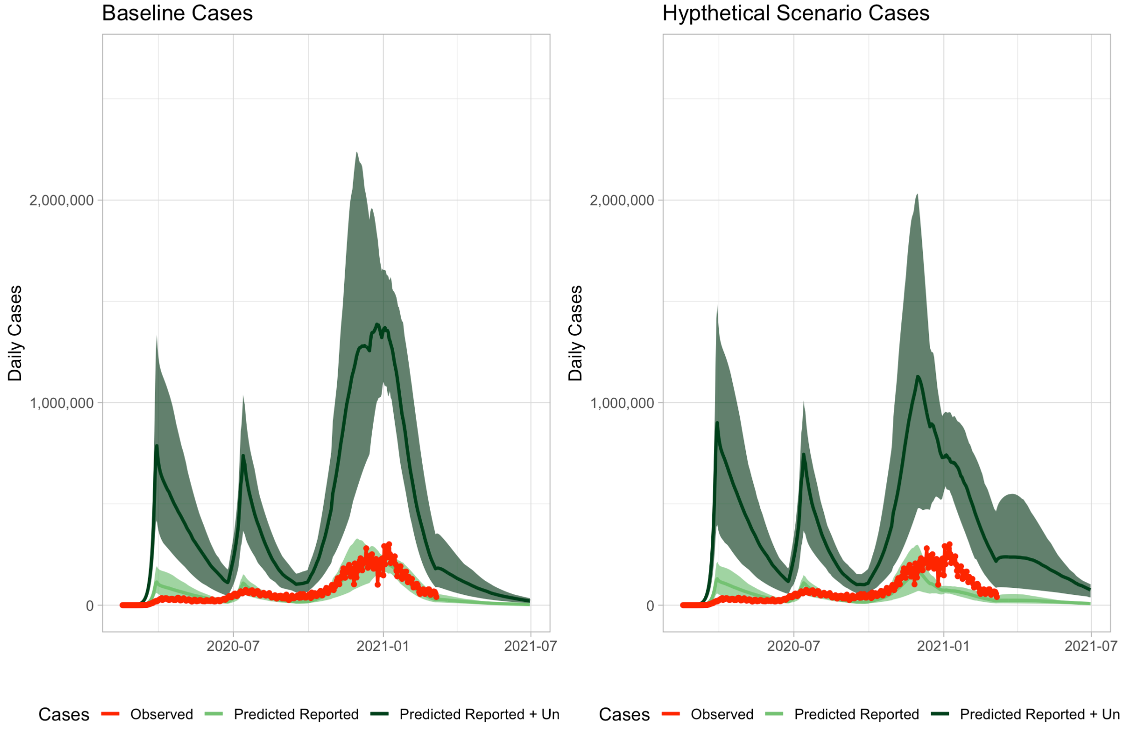

The above graphs show the Predicted Reported + Unreported cases over time expected from the Baseline compared with the Hypothetical scenario.

Cumulative mortality

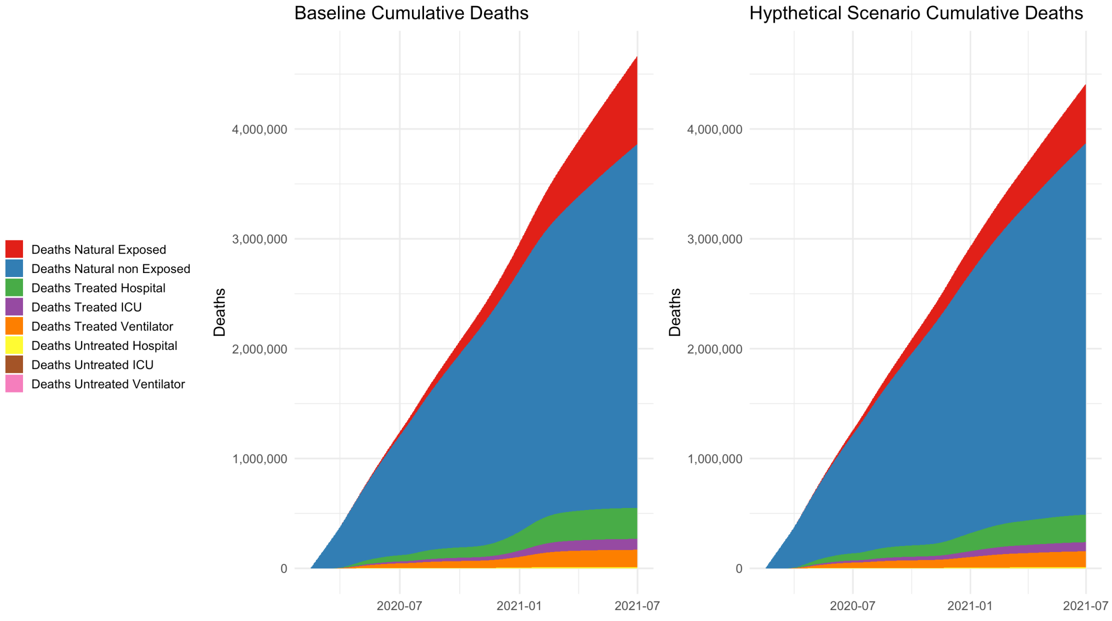

The above graphs show the cumulative mortality over time expected from the Baseline compared with the Hypothetical scenario. The expected mortality has been further

stratified to reflect death from severe COVID-19 during hospital treatment or in severely ill patients who were not able to access these resources. The predicted COVID-19 attributable deaths may be compared with mortality from other causes where patients may or may not test positive for COVID-19 post-mortem depending on their infection status upon death.

Deaths per Age Category

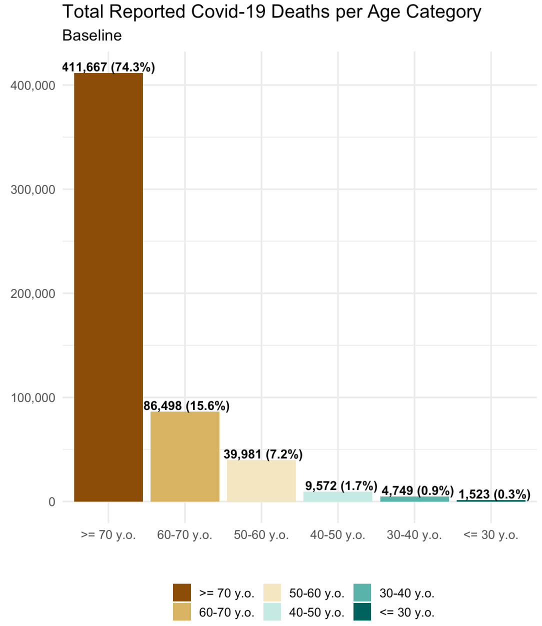

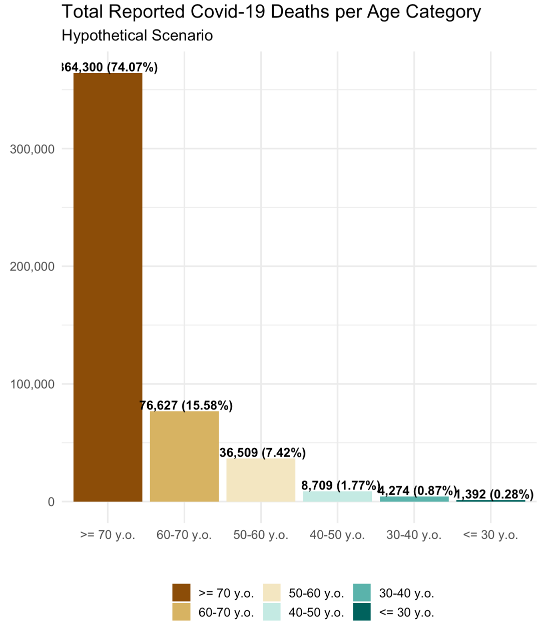

Hospital occupancy

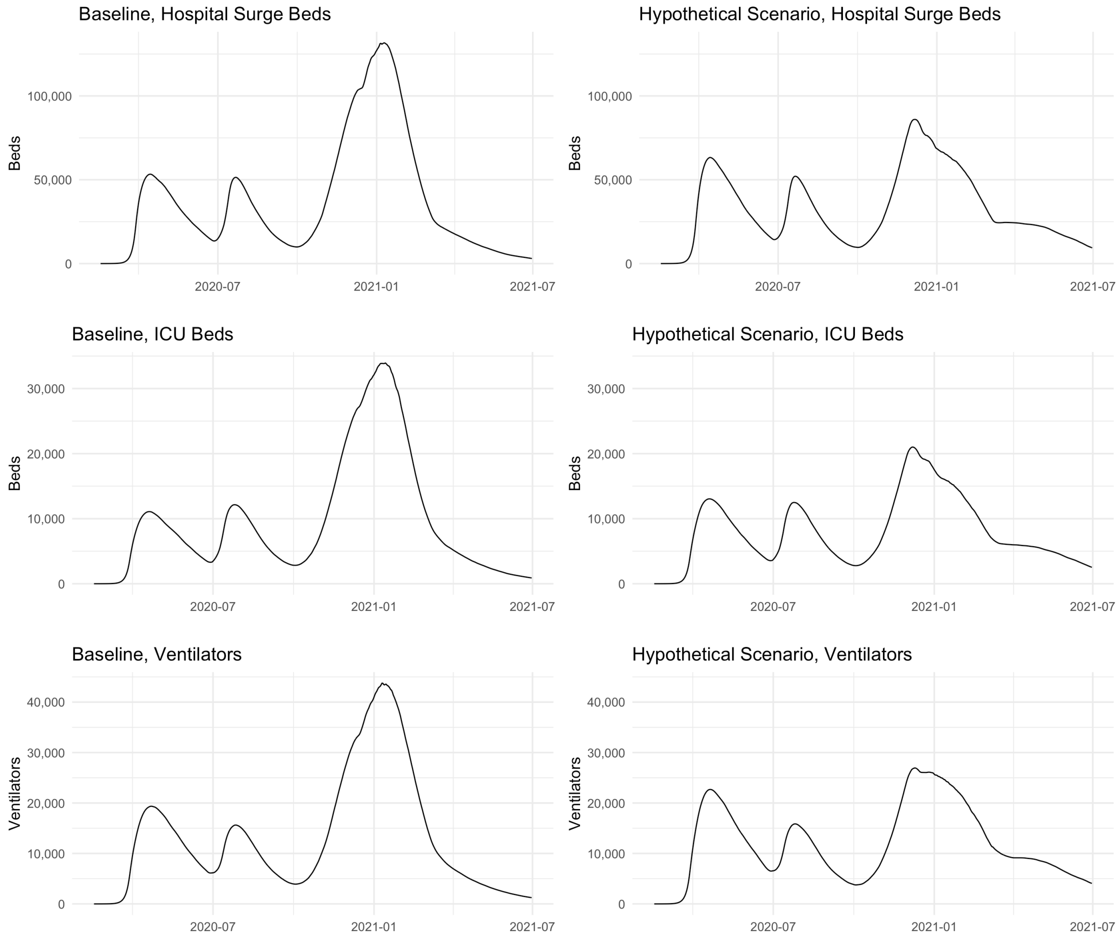

The above graphs show expected hospital occupancy for the baseline and hypothetical scenarios depending on the input values of resource availability as follows:

- Maximum number of hospital surge beds is 800685
- Maximum number of ICU beds without ventilators is 60372
- Maximum number of ICU beds with ventilators is 60000

In most simulations, the occupancy for each classification will plateau at the input capacity. A level of flexibility is assumed in the model which allows for the occupancy to exceed capacity in some cases. Especially in the hospital surge category. For this reason, some simulations will predict occupancy exceeding capacity.

Hospital demand

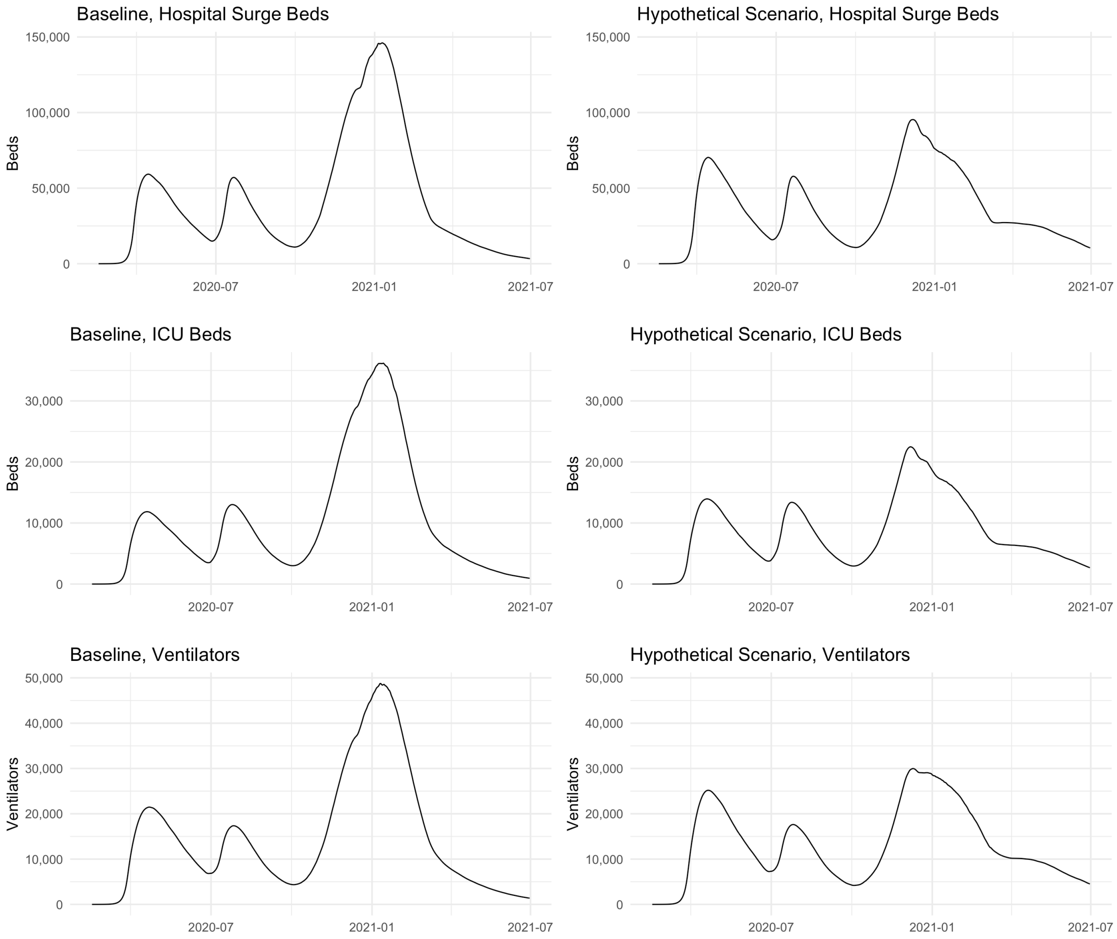

The above graphs represent the expected demand for baseline and hypothetical scenarios for hospital surge beds, ICU beds without ventilators, and ICU beds with ventilators, even if some of this demand cannot be met with the current health system.

Reproduction number

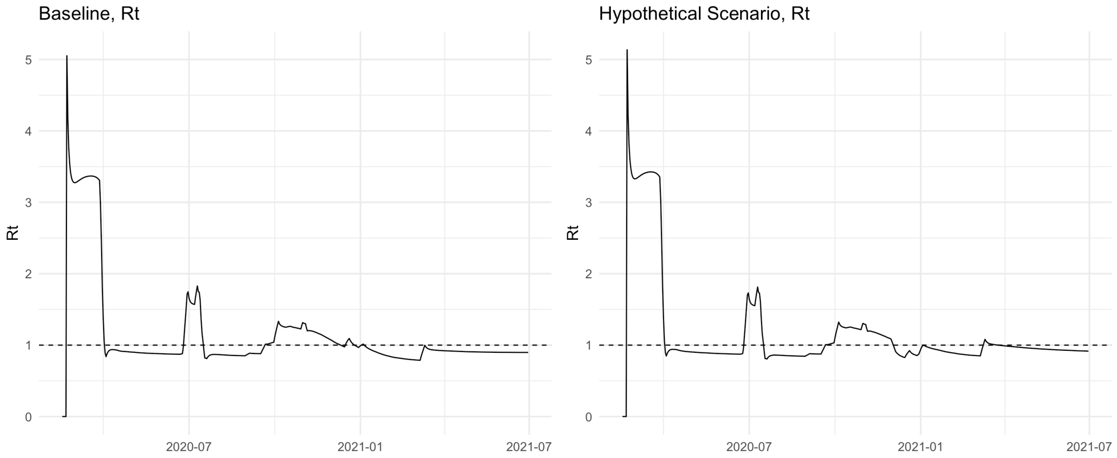

The above graphs show the model prediction of the reproduction number over time, R_t, if this value is greater than 1, then the epidemic may spread in the population. R_t may be less than 1 if either the interventions deployed are sufficient to stop transmission, if a sufficient percentage of the population have been exposed to the infection and are immune (herd immunity), or some combination of these outcomes.

References

1. Prem, K., A.R. Cook, and M. Jit, Projecting social contact matrices in 152 countries using contact surveys and demographic data. PLoS Comput Biol, 2017. 13(9): p. e1005697.

2. Henrik Salje, Cécile Tran Kiem, Noémie Lefrancq, Noémie Courtejoie, Paolo Bosetti, Juliette Paireau, Alessio Andronico, Nathanaël Hozé, Jehanne Richet, Claire-Lise Dubost, Yann Le Strat, Justin Lessler, Daniel Levy-Bruhl, Arnaud Fontanet, Lulla Opatowski, Pierre-Yves Boelle, and Simon Cauchemez. Estimating the burden of sars-cov-2 in france. Science, 369(6500):208–211, 2020.

3. Lapidus, N., Zhou, X., Carrat, F. *et al.* Biased and unbiased estimation of the average length of stay in intensive care units in the Covid-19 pandemic. *Ann. Intensive Care* **10,** 135 (2020). https://doi.org/10.1186/s13613-020-00749-6

4. Epic Health Research Network, https://ehrn.org/articles/inpatient-lengths-of-stay-and-number-of-icu-days-among-covid-19-patients-differ-from-common-model-assumptions/

Appendix: Parameter Table

Sheet-Intervention	Parameter	Value
Parameters	Number of exposed people at start date	100
Parameters	Proportion of population with partial immunity at the start date	0
Parameters	Probability of infection given contact (0 to 0.2)	0.031
Parameters	Percentage of all asymptomatic infections that are reported	2
Parameters	Percentage of all symptomatic infections that are reported	75
Parameters	Percentage of denied hospitalisations that are reported	90
Parameters	Percentage of non-severe hospitalisations that are appropriately treated	90
Parameters	Percentage of severe hospitalisations that are appropriately treated	90
Parameters	Percentage of all asymptomatic infections in previously vaccinated people that are reported	0
Parameters	Percentage of all asymptomatic infections in previously vaccinated and exposed people that are reported	0
Parameters	Percentage of all asymptomatic infections in previously infected people that are reported	0
Parameters	Percentage of all symptomatic infections in previously vaccinated people that are reported	0
Parameters	Percentage of all symptomatic infections in previously vaccinated and exposed people that are reported	0
Parameters	Percentage of all symptomatic infections in previously infected people that are reported	0
Parameters	Percentage of all people dying outside the hospital with asymptomatic infections reported as covid-deaths	0

Parameters	Percentage of all people dying outside the hospital with symptomatic infections reported as covid-deaths	10
Parameters	Percentage of all people dying outside the hospital with severe infections reported as covid-deaths	10
Parameters	Iterations (1 to 10,000)	100
Parameters	Noise (0.01 to 0.2)	0.03
Parameters	Confidence (5 to 25)	5
Parameters	Average sample size for seroprevalence	100
Country Area Param	Social Contacts Data	United States of America
Country Area Param	Mean Household size	2.5
Country Area Param	Mean number of infectious migrants per day	3.5
Virus Param	Relative infectiousness of incubation phase	50
Virus Param	Average incubation period (1 to 7 days)	3.5
Virus Param	Average duration of symptomatic infection period (1 to 7 days)	4.5
Virus Param	Month of peak infectivity of the virus (1, 2, …, 12)	January
Virus Param	Annual variation in infectivity of the virus	0
Virus Param	Average duration of immunity (0.5 to 150)	150
Virus Param	Probability upon infection of developing clinical symptoms	15
Virus Param	Probability upon hospitalisation of requiring ICU admission	25
Virus Param	Probability upon admission to the ICU of requiring a ventilator	60
Virus Param	Proportion of hospitalised patients needing O2	50
Virus Param	Probability upon infection of developing clinical symptoms if previously vaccinated	15

Virus Param	Probability upon infection of developing clinical symptoms if previously vaccinated and exposed	15
Virus Param	Probability upon infection of developing clinical symptoms if previously infected	15
Virus Param	Probability upon hospitalisation of requiring ICU admission if previously vaccinated	25
Virus Param	Probability upon hospitalisation of requiring ICU admission if previously vaccinated and exposed	25
Virus Param	Probability upon hospitalisation of requiring ICU admission if previously infected	25
Virus Param	Probability upon admission to the ICU of requiring a ventilator if previously vaccinated	60
Virus Param	Probability upon admission to the ICU of requiring a ventilator if previously vaccinated and exposed	60
Virus Param	Probability upon admission to the ICU of requiring a ventilator if previously infected	60
Virus Param	Probability of infection of people that have recovered from a previous infection	0
Virus Param	Change in probability of requiring hospitalisation if previously vaccinated	95
Virus Param	Change in probability of requiring hospitalisation if previously infected	95
Virus Param	Change in probability of requiring hospitalisation if previously infected and vaccinated	95
Virus Param	Days from seropositve to seronegative	100
Hospitalisation Param	Maximum number of hospital surge beds	800685
Hospitalisation Param	Maximum number of ICU beds without ventilators	60372
Hospitalisation Param	Maximum number of ICU beds with ventilators	60000

Hospitalisation Param	Relative percentage of regular daily contacts when hospitalised:	5
Hospitalisation Param	Scaling factor for infection hospitalisation rate: (0.1 to 5)	1
Hospitalisation Param	Probability of dying when hospitalised (not req O2):	15
Hospitalisation Param	Probability of dying when hospitalised if req O2:	25
Hospitalisation Param	Probability of dying when denied hospitalisation (not req O2):	30
Hospitalisation Param	Probability of dying when denied hospitalisation if req O2:	35
Hospitalisation Param	Probability of dying when admitted to ICU (not req O2):	40
Hospitalisation Param	Probability of dying when admitted to ICU if req O2:	50
Hospitalisation Param	Probability of dying when admission to ICU denied (not req O2):	60
Hospitalisation Param	Probability of dying when admission to ICU denied if req O2:	70
Hospitalisation Param	Probability of dying when ventilated:	80
Hospitalisation Param	Probability of dying when ventilator denied:	95
Hospitalisation Param	Probability of dying when ventilator required and not going to hospital:	100
Hospitalisation Param	Probability of dying when icu required (not O2) and not going to hospital:	80
Hospitalisation Param	Probability of dying when icu required (req O2) and not going to hospital:	95
Hospitalisation Param	Duration of hospitalised infection: (1 to 30)	6
Hospitalisation Param	Duration of ICU infection: (1 to 30)	10
Hospitalisation Param	Duration of ventilated infection: (1 to 30)	12

Self-isolation if Symptomatic	Adherence:	66
(*Self-isolation) Screening	Overdispersion: (1, 2, 3, 4 or 5)	4
(*Self-isolation) Screening	Test Sensitivity:	80
(*Self-isolation) Household Isolation	Days in isolation for average person:	14
(*Self-isolation) Household Isolation	Days to implement maximum quarantine coverage: (1 to 5)	2
(*Self-isolation) Household Isolation	Decrease in the number of other contacts when quarantined:	20
(*Self-isolation) Household Isolation	Increase in the number of contacts at home when quarantined:	100
Sheet-Intervention	Parameter	Value
——————————————	———-	——————————————
Parameters	Number of exposed people at start date	100
Parameters	Proportion of population with partial immunity at the start date	0
Parameters	Probability of infection given contact (0 to 0.2)	0.031
Parameters	Percentage of all asymptomatic infections that are reported	2
Parameters	Percentage of all symptomatic infections that are reported	75
Parameters	Percentage of denied hospitalisations that are reported	90
Parameters	Percentage of non-severe hospitalisations that are appropriately treated	90
Parameters	Percentage of severe hospitalisations that are appropriately treated	90
Parameters	Percentage of all asymptomatic infections in previously vaccinated people that are reported	0

Parameters	Percentage of all asymptomatic infections in previously vaccinated and exposed people that are reported	0
Parameters	Percentage of all asymptomatic infections in previously infected people that are reported	0
Parameters	Percentage of all symptomatic infections in previously vaccinated people that are reported	0
Parameters	Percentage of all symptomatic infections in previously vaccinated and exposed people that are reported	0
Parameters	Percentage of all symptomatic infections in previously infected people that are reported	0
Parameters	Percentage of all people dying outside the hospital with asymptomatic infections reported as covid-deaths	0
Parameters	Percentage of all people dying outside the hospital with symptomatic infections reported as covid-deaths	10
Parameters	Percentage of all people dying outside the hospital with severe infections reported as covid-deaths	10
Parameters	Iterations (1 to 10,000)	100
Parameters	Noise (0.01 to 0.2)	0.03
Parameters	Confidence (5 to 25)	5
Parameters	Average sample size for seroprevalence	100
Country Area Param	Social Contacts Data	United States of America
Country Area Param	Mean Household size	2.5
Country Area Param	Mean number of infectious migrants per day	3.5
Virus Param	Relative infectiousness of incubation phase	50
Virus Param	Average incubation period (1 to 7 days)	3.5
Virus Param	Average duration of symptomatic infection period (1 to 7 days)	4.5

Virus Param	Month of peak infectivity of the virus (1, 2, …, 12)	January
Virus Param	Annual variation in infectivity of the virus	0
Virus Param	Average duration of immunity (0.5 to 150)	150
Virus Param	Probability upon infection of developing clinical symptoms	15
Virus Param	Probability upon hospitalisation of requiring ICU admission	25
Virus Param	Probability upon admission to the ICU of requiring a ventilator	60
Virus Param	Proportion of hospitalised patients needing O2	50
Virus Param	Probability upon infection of developing clinical symptoms if previously vaccinated	15
Virus Param	Probability upon infection of developing clinical symptoms if previously vaccinated and exposed	15
Virus Param	Probability upon infection of developing clinical symptoms if previously infected	15
Virus Param	Probability upon hospitalisation of requiring ICU admission if previously vaccinated	25
Virus Param	Probability upon hospitalisation of requiring ICU admission if previously vaccinated and exposed	25
Virus Param	Probability upon hospitalisation of requiring ICU admission if previously infected	25
Virus Param	Probability upon admission to the ICU of requiring a ventilator if previously vaccinated	60
Virus Param	Probability upon admission to the ICU of requiring a ventilator if previously vaccinated and exposed	60
Virus Param	Probability upon admission to the ICU of requiring a ventilator if previously infected	60

Virus Param	Probability of infection of people that have recovered from a previous infection	0
Virus Param	Change in probability of requiring hospitalisation if previously vaccinated	95
Virus Param	Change in probability of requiring hospitalisation if previously infected	95
Virus Param	Change in probability of requiring hospitalisation if previously infected and vaccinated	95
Virus Param	Days from seropositve to seronegative	100
Hospitalisation Param	Maximum number of hospital surge beds	800685
Hospitalisation Param	Maximum number of ICU beds without ventilators	60372
Hospitalisation Param	Maximum number of ICU beds with ventilators	60000
Hospitalisation Param	Relative percentage of regular daily contacts when hospitalised:	5
Hospitalisation Param	Scaling factor for infection hospitalisation rate: (0.1 to 5)	1
Hospitalisation Param	Probability of dying when hospitalised (not req O2):	15
Hospitalisation Param	Probability of dying when hospitalised if req O2:	25
Hospitalisation Param	Probability of dying when denied hospitalisation (not req O2):	30
Hospitalisation Param	Probability of dying when denied hospitalisation if req O2:	35
Hospitalisation Param	Probability of dying when admitted to ICU (not req O2):	40
Hospitalisation Param	Probability of dying when admitted to ICU if req O2:	50
Hospitalisation Param	Probability of dying when admission to ICU denied (not req O2):	60

Hospitalisation Param	Probability of dying when admission to ICU denied if req O2:	70
Hospitalisation Param	Probability of dying when ventilated:	80
Hospitalisation Param	Probability of dying when ventilator denied:	95
Hospitalisation Param	(v16.2) Probability of dying when ventilator required and not going to hospital:	100
Hospitalisation Param	(v16.2) Probability of dying when icu required (not O2) and not going to hospital:	80
Hospitalisation Param	(v16.2) Probability of dying when icu required (req O2) and not going to hospital:	95
Hospitalisation Param	Duration of hospitalised infection: (1 to 30)	6
Hospitalisation Param	Duration of ICU infection: (1 to 30)	10
Hospitalisation Param	Duration of ventilated infection: (1 to 30)	12
Self-isolation if Symptomatic	Adherence:	66
(*Self-isolation) Screening	Overdispersion: (1, 2, 3, 4 or 5)	4
(*Self-isolation) Screening	Test Sensitivity:	80
(*Self-isolation) Household Isolation	Days in isolation for average person:	14
(*Self-isolation) Household Isolation	Days to implement maximum quarantine coverage: (1 to 5)	2
(*Self-isolation) Household Isolation	Decrease in the number of other contacts when quarantined:	20
(*Self-isolation) Household Isolation	Increase in the number of contacts at home when quarantined:	100
Social Distancing	Adherence:	80
Handwashing	Efficacy: (0-25%)	10
Mask Wearing	Efficacy: (0-35%)	20
Working at Home	Efficacy:	85

Working at Home	Home contacts inflation due to working from home:	10
School Closures	Home contacts inflation due to school closure:	20
Shielding the Elderly	Efficacy:	95
Shielding the Elderly	Minimum age for elderly shielding: (0 to 100)	70
Vaccination	Time to reach target coverage (1 to 52)	4
Vaccination	(v16.2) Duration of efficacious period	100
Vaccination	(v16.2) Duration of efficacious period if previously infected	100
Vaccination	Efficacy	5
Vaccination	(v16.2) Efficacy if previously infected	100
Mass Testing	Sensitivity	80
Mass Testing	Isolation days	14
Dexamethasone	Relative risk of dying if needing O2 and taking Dex	82
Dexamethasone	Relative risk of dying if needing ventilation and taking Dex	64
Dexamethasone	Relative risk of dying if needing but not receiving O2 and taking Dex	82
Dexamethasone	Relative risk of dying if needing but not receiving ventilation and taking Dex	64
Dexamethasone	Change in ventilation requirement if given Dex	87

3. Disclaimer for CoMo Consortium Model

(from the model website, https://comomodel.net/)

"Whilst every effort has been taken during the development of this tool/model for it to be as accurate and reliable as possible it is important that the user understands that the outputs are a prediction based on the assumptions chosen through the input parameter values. In view of the current uncertainty on the COVID-19 mechanisms of action, the output of the model should be used to explore multiple scenarios and in combination with a larger evidence base during decision-making. The appropriate use of this tool/model and its output can contribute to effective policymaking, but misuse or misinterpretation of the output can mislead decision-making. Any decisions taken whist using these tools are the responsibility of the user and no liability whatsoever will be taken by the developers/authors of the tool"
